# Advances and Recent Trends in Heterogeneous Photo(Electro)-Catalysis for Solar Fuels and Chemicals

**DOI:** 10.3390/molecules20046739

**Published:** 2015-04-15

**Authors:** James Highfield

**Affiliations:** Heterogeneous Catalysis, Institute of Chemical & Engineering Sciences (ICES, A * Star), 1 Pesek Road, Jurong Island, 627833, Singapore; E-Mail: james_highfield@ices.a-star.edu.sg; Tel.: +65-6796-3805; Fax: +65-6316-6188

**Keywords:** solar fuels, heterogeneous photocatalysis, water splitting, renewable hydrogen, visible sensitization, hydrogen peroxide, bio-oxygenates photoreforming, electrocatalysis, carbon dioxide reduction

## Abstract

In the context of a future renewable energy system based on hydrogen storage as energy-dense liquid alcohols co-synthesized from recycled CO_2_, this article reviews advances in photocatalysis and photoelectrocatalysis that exploit solar (photonic) primary energy in relevant endergonic processes, viz., H_2_ generation by water splitting, bio-oxygenate photoreforming, and artificial photosynthesis (CO_2_ reduction). Attainment of the efficiency (>10%) mandated for viable techno-economics (USD 2.00–4.00 per kg H_2_) and implementation on a global scale hinges on the development of photo(electro)catalysts and co-catalysts composed of earth-abundant elements offering *visible-light-driven* charge separation and surface redox chemistry in high quantum yield, while retaining the chemical and photo-stability typical of titanium dioxide, a ubiquitous oxide semiconductor and performance “benchmark”. The dye-sensitized TiO_2_ solar cell and multi-junction Si are key “voltage-biasing” components in hybrid photovoltaic/photoelectrochemical (PV/PEC) devices that currently lead the field in performance. Prospects and limitations of visible-absorbing particulates, e.g., nanotextured crystalline α-Fe_2_O_3_, g-C_3_N_4_, and TiO_2_ sensitized by C/N-based dopants, multilayer composites, and plasmonic metals, are also considered. An interesting trend in water splitting is towards hydrogen peroxide as a solar fuel and value-added green reagent. Fundamental and technical hurdles impeding the advance towards pre-commercial solar fuels demonstration units are considered.

## 1. Introduction

In 2004 Nobel Laureate Richard Smalley, discoverer of fullerenes and pioneer of modern nanoscience, gave a talk on the 10 great challenges facing humanity in the 3rd millenium. Placing *energy* at the top of the list, he proposed divestiture of fossil fuels in favour of sustainable and environmentally benign alternatives [1]. He also envisioned that nanotechnology, *i.e.*, the design and assembly of nanometer scale structures, would play a key role in our future prosperity. In fact, it can be argued that nanomaterials have already been exploited for many years in the form of heterogeneous catalysts in industrial chemical processing, responsible for the rapid growth in civilized life during the 20th century. Much of this was done with only marginal understanding of their workings prior to the advent of *in-situ* surface characterization, high-resolution imaging tools, and theoretical (computational) modeling [2,3]. Nowadays, the development of nanocatalysts with functionality optimized “by rational design” is a popular theme [4,5,6,7,8] but it remains an uphill challenge due to the myriad complexity of catalytic phenomena [9]. Nonetheless, recent articles focusing on the prospects for such materials in renewable energy applications give some ground for optimism [10,11,12]. They also introduce the main topic of this report, viz., the prodigious growth in research into heterogeneous photocatalysis, as attested by the drastic increase in literature citations over the last 20 years. While many of these concern environmental or “advanced oxidation” applications [13], multitudinous examples related to energy topics can be located under key (search) phrases like solar fuels, photo-catalytic hydrogen, photo-reforming, water photo-splitting, CO_2_ photo-reduction, *etc.* A photocatalyst can be defined as “a solid material that accelerates a chemical reaction by light absorption while itself remaining unchanged” [14]. Just as in a thermal heterogeneous catalyst, a chemical process is made faster due to a significant lowering of the energy barrier of the associated transition state, e.g., by providing surface sites that activate unique modes of adsorption. On a typical semiconductor photocatalyst like titanium dioxide (TiO_2_), the adsorbed (dark) state is further activated (or entirely new states are created) by surface interaction with highly energetic photo-induced charges, viz., electrons (e^−^) and holes (h^+^). Band-gap excitation of TiO_2_ (λ ≤ 400 nm) creates photons with an energy ≥300 kJ/mol. In principle, the use of photocatalysts enables substitution of expensive process heat by cheap solar (photon) energy, leading to reduced operating costs. However, an added impetus for research is that they can also drive *endergonic* (thermodynamically uphill) processes by converting light energy into stored chemical energy (bond enthalpy). In other words, the reaction selectivity can be steered towards more useful products. By analogy with the natural process, when the reactants are water and carbon dioxide, this is sometimes referred to as *artificial photosynthesis* [15], in which H_2_ and/or its reaction product with CO_2_ is isolated as a *solar fuel*.

To better appreciate the future impact of any viable solar fuel technology, it is helpful to consider it in the broader context of renewable energy schemes and their current limitations. In principle, the ideal energy carrier from an energetic and environmental viewpoint is hydrogen. On a weight basis, it has the highest energy density of any fuel (143 MJ/kg, or 3× the value of gasoline) and it burns cleanly and efficiently to water, producing heat and/or electrical power in a fuel cell [16]. Unfortunately, H_2_ also has the lowest volumetric energy density under ambient conditions (0.011 MJ/L), making its storage in physical form (compressed, liquefied, adsorbed, *etc.*) impractical and expensive, especially for applications in the transportation sector [17]. Any renewable fuel should retain the positive attributes of gasoline but offer the environmental benefits of H_2_. It must be a liquid (for ease of handling) and have a practical energy density (≥20 MJ/L). It should also contain a substantial level of “incipient hydrogen” (≥12 wt %) and be carbon-neutral in the long term. The recent (transitional) strategy of fuel “decarbonization” aims to exploit the clean energy associated with the H-component in fossil fuels as these become depleted [18]. 

**Figure 1:**
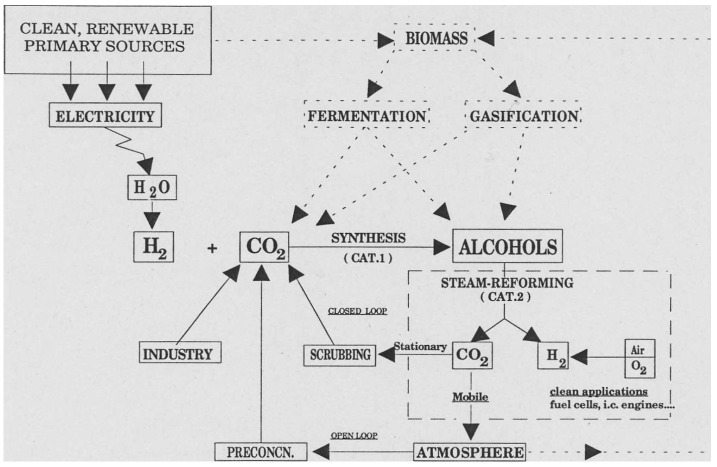
Idealized energy scheme based on renewable H_2_ stored in simple alcohols (reproduced from [19] with permission).

A previous review by this author [19] dealt almost exclusively with *thermal* heterogeneous catalytic processes in a future renewable hydrogen energy system schematized in Figure 1.

In a gas phase catalytic process, H_2_ derived from renewable primary sources (solar-electric, hydro-electric, *etc.*) is converted to alcohol(s) by a synthesis reaction with CO_2_, itself recycled from industrial sources in concentrated form (closed loop). In the near future, the more challenging (open loop) process, *i.e.*, direct capture of CO_2_ from the atmosphere, may become technical reality [20,21]. Simple alcohols are ideal H-carriers as they can be synthesized quite selectively while H_2_ can be released at relatively low temperature. Insofar as biomass can supply renewable H_2_, alcohols and oxygenates (even sugars) by gasification, fermentation, carbohydrate hydrolysis, *etc.* [22], this natural renewable (CO_2_-neutral) energy source is integrated into the overall scheme. Essentially the same scientific precepts have since been advanced in a monograph by Olah *et al.*, which focuses on storage solely as methanol since this is already technically feasible [23]. The current status of *The Methanol Economy* has been the subject of a recent review, which re-emphasizes that the price of the alcohol will be strongly dependent on the cost of renewable electricity for H_2_ generation [24]. Cost analyses show that “renewable” methanol will probably be 2–3× more expensive than the methane-based commodity [25] (see also Section 7). As regards the main catalytic cycle, the alcohol(s) synthesis can be written in general form as:

n CO_2_ + 3n H_2_ ↔ C_n_H_2n+1_OH + (2n-1) H_2_O (1)

Under high pressure, reaction (1) is favoured (∆G < 0) at ambient temperature but synthesis catalysts generally operate above 220 °C due to kinetic limitations. Product selectivity in ethanol synthesis is a major challenge [26,27]. Upon demand, H_2_ is released catalytically from aqueous alcohol vapours to generate heat and power via steam-reforming (SR):

C_n_H_2n+1_OH + (2n-1) H_2_O ↔ n CO_2_ + 3n H_2_ (2)

SR is an endothermic process (∆H ≥ 0) but reaction (2) is theoretically feasible (∆G ≤ 0) near ambient for methanol, and above 230 °C for ethanol due to the molar volume increase (entropy factor). Since SR is simply the reverse of synthesis, catalysts for both reactions are similar. Formulations based on Cu (modified by Co, Ni, Pd, Rh, *etc.*) on various oxide supports are quite effective. The main technical hurdle is the need for “fuel processing” because low levels of carbon monoxide in the reformate poison the fuel cell (Pt) anode and must be eliminated [28,29]. In addition, although it has been a growing area of research in the last 20 years, ethanol SR still suffers from catalyst deactivation due to carbon deposition, probably linked to the high temperatures necessary to establish good conversion rates [30,31]. Nevertheless, the scope for bio-hydrogen resources now encompasses a range of oxygenates and polyols, e.g., glycerol (a waste product from bio-diesel synthesis [32,33]), in which coking can be minimized via aqueous-phase-reforming (APR) under mild conditions [34].

For maximum technical impact, photocatalysis should logically be applied to the most energy-demanding steps in the scheme under consideration. Artificial photosynthesis to create a fuel such as methanol from aqueous CO_2_:

CO_2_ + 2 H_2_O ↔ CH_3_OH + 1.5 O_2_ (3)

is highly endothermic (∆H = +727 kJ/mol) but can be driven by solar photons in the visible/near IR region because it is 6 e^−^ process. Since reaction (3) involves water splitting (H abstraction) implicitly, it effectively couples two stages in the above scheme (H_2_ generation and methanol synthesis), offering a potential process simplification. Supplying the energy needed for (endothermic) steam-reforming of alcohols (∆H° ≈ +130 kJ/mol CH_3_OH or +175 kJ/mol C_2_H_5_OH) by means of photons is another obvious prospect, giving rise to intensive recent interest in *photo-reforming* [35]. In the review that follows, space limitations and a plethora of recent review articles [10,11,36,37,38,39,40,41,42,43,44,45,46,47,48,49,50,51,52,53,54,55,56] act as major con-straints on detail and impose a necessarily high degree of selectivity in given examples. Similarly, the emerging field of “renewable fuels from CO_2_ and H_2_O by solar-*thermal* processes” is beyond its scope [57]. As the title implies, this review deals only with materials that respond to (absorb) a significant fraction of the solar power spectrum, over 90% of which lies in the visible and near infrared region. Thus, recent advances in “sensitization” methods for TiO_2_, the benchmark photocatalyst, are covered in some depth along with exploration of stable and non-toxic semiconductors of more suitable bandgap, either for independent use or in tandem (composite) arrangement for improved efficiency. The author has endeavoured to strike a balance between topicality and novelty, and apologizes in advance to the many authors who are not cited directly. Advances in selected materials are given regardless of their testing configuration, be it as dispersed nanoparticles in suspension or as “wired” electrodes in a photoelectrochemical cell (PEC). The question of the degree of complexity of any solar fuel “device”, its future amenability to scale-up, and ultimate impact on process techno-economics (fuel cost) is still at an early stage of evaluation [58,59,60,61,62]. This is considered near the end of the review, along with the inevitable trend towards devices composed of “earth-abundant” elements [62,63,64,65,66,67,68], as necessitated by the ultimate (global) scale of fossil fuels replacement (see Section 7).

## 2. Water Splitting and Target Efficiency in Solar Hydrogen Generation

The fundamental groundwork in evaluating the maximum efficiency achievable in a solar photonic device was done by Bolton and co-workers [69,70,71], taking water splitting as the model reaction:

H_2_O → H_2_ + 0.5 O_2_ (4)

**Figure 2:**
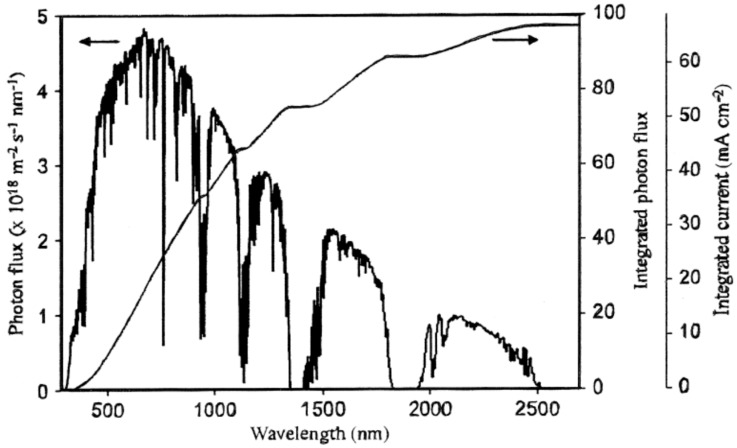
Solar photon flux at the earth’s surface *vs.* wavelength and integrated current density obtainable from an ideal PV cell (adapted from [72] with permission of Elsevier).

This two-electron, or effectively two-photon (one photon per H atom), reduction process is highly energetic and thermodynamically uphill by 237 kJ/mol H_2_ or 119 kJ/mol photons, corresponding to a wavelength of 1008 nm (1.23 eV—all redox potentials are given *vs.* the normal hydrogen electrode at pH = 0). Inspection of the solar spectrum in Figure 2 shows that photons of greater energy (λ ≤ 1008 nm) constitute only one half of the total incident solar flux. 

**Figure 3:**
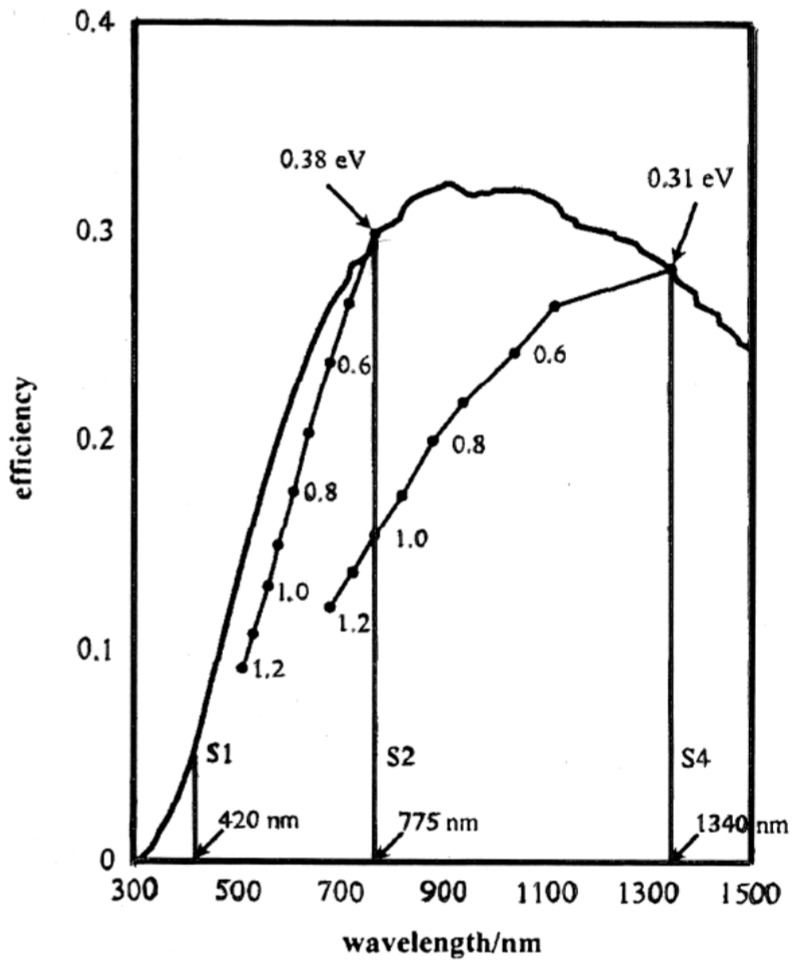
Solar photon-to-chemical energy conversion efficiency in a single bandgap current device (adapted from [71] with permission of Elsevier).

**Figure 4:**
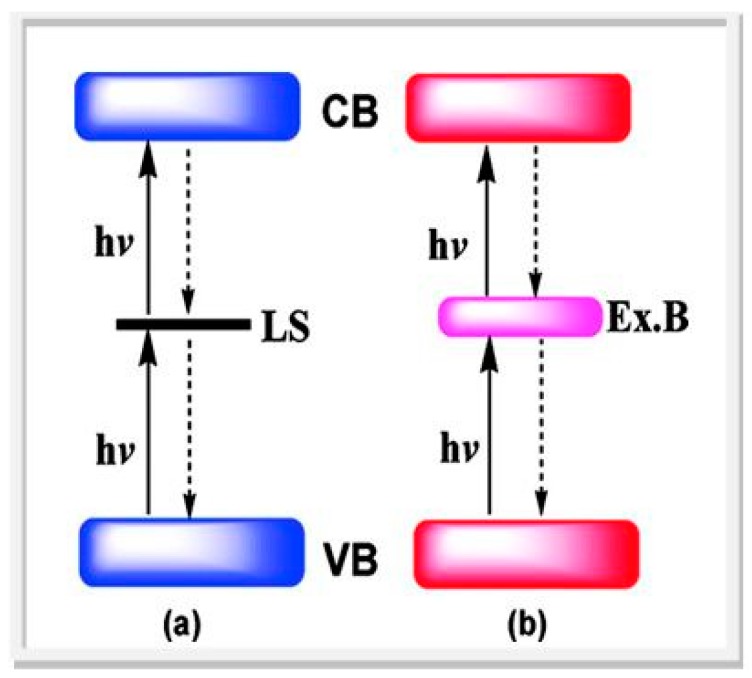
Successive two-photon excitation (**a**) via localized dopant states (LS) (**b**) via delocalized states in an extrinsic band (Ex.B) (reproduced from [74] with permission of ACS.

Furthermore, not all the energy in these photons is available for chemical energy storage after absorption due to intraband *thermalization*, *i.e.*, ultra-fast relaxation from excited vibrational states of the first electronic excited state (or conduction band in a semiconductor). To compensate for this energy loss the wave-length threshold must shift to 775 nm in this so-called S2 process, as shown in Figure 3. When entropic losses (non-equivalence of internal energy and Gibbs energy) are also factored in, the maximum efficiency (η_STH_) of a device for solar-to-hydrogen (STH) energy conversion based on a single photo-system is around 30%. Also shown in Figure 3 are plots with more realistic entropic losses (>0.4 eV) and their progressive erosion of efficiency. In principle, a 4 e^−^ (S4) process extends the useful spectral range to 1340 nm based on successive absorption of two low energy photons to drive a single electron event like proton discharge (H^+^ + e^−^ → H**∙**). For a semiconductor, this is a futuristic concept because it would require hypothetical long-lived mid-gap states to be populated in the first photon absorption event, as shown in Figure 4. This will require a major advance in “bandgap engineering”, but may yield so-called 3rd generation photocatalysts (*vide infra*) [73,74]. 

**Figure 5:**
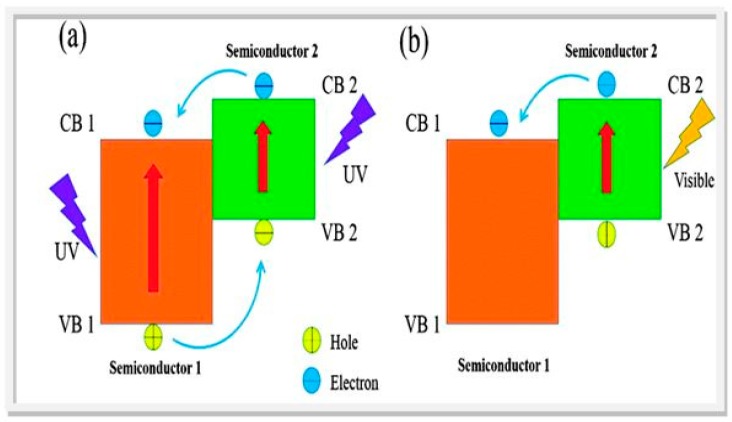
Dual absorbers with complementary bandgap/band edge positions (type II heterojunction) under (**a**) UV; and (**b**) Visible excitation (reproduced from [82] with permission of PCCP Owner Societies).

On the other hand, coupling of two absorbers of complementary bandgap and band-edge position (type II -see Figure 5) each absorbing two photons in a dual (D4) or tandem device is a viable water splitting configuration and offers better matching to the solar spectrum [75,76]. In practice, the mechanistic complexity (kinetic barrier) in the water oxidation half-cell reaction has kept conversion efficiencies below 2% until recently [60,61,62,77,78]. Efficiencies in the more challenging process of CO_2_ photo-reduction are still below 0.5% [79]. The minimum workable efficiency for implementation of any solar-to-hydrogen (STH) process is taken as 10%, leading to a H_2_ price approaching the DOE target of $4.00 per kg [60]. Immediately obvious from Figure 2 and Figure 3 is that pristine TiO_2_, the prototypical semiconductor photocatalyst [80,81] with a band-gap of ~3 eV (λ ≤ 400 nm), absorbs just 4% of solar light. However, since this oxide is cheap and non-toxic and has otherwise excellent material properties, suitable energetics (band edge positions) to drive both proton reduction and water oxidation, stability in aqueous environment, *etc.*, ways of *sensitizing* TiO_2_ to visible light are being studied intensively. At the same time, other non-TiO_2_-based systems with intrinsic visible absorption are urgently sought.

Incorporation of materials that perform efficient solar light harvesting is a fundamental (thought not the only) pre-condition for an effective energy conversion device. While it is generally assumed that absorption of one photon of energy exceeding the bandgap creates one electron/hole pair (exciton) with 100% efficiency, the fate of these excited states is less clear. One advantage of screening photocatalysts in “wired” mode, e.g., as photoanodes in an electrochemical cell, is that it promotes better charge separation via “band-bending”, viz., the space-charge (depletion) layer formed spontaneously at the semiconductor/liquid junction. It also facilitates measurement of the combined efficiency of the two key initiatory processes (light absorption and charge collection) in terms of the resulting photocurrent. The incident photon-to-current conversion efficiency (IPCE) is a figure of merit for any photovoltaic (PV) cell. The photocurrent density limit shown in Figure 2 (~65 mA·cm^−2^) is the ideal value achievable under so-called AM1.5G (1 Sun) illumination, corresponding to an incident optical power close to 1000 W·m^−2^ or 100 mW·cm^−2^ [72,83]. In solar fuel generation, these photo-generated charges are required instead to drive surface redox chemistry, in which the presence of suitable co-catalysts and rapid turnover of the substrate (diffusion) are crucial to performance. Due to these kinetic limitations, STH efficiencies lie below the PV (IPCE) value.

## 3. Advances in Absorber Materials with Improved Solar Spectral Matching

### 3.1. Modified TiO_2_

**Figure 6:**
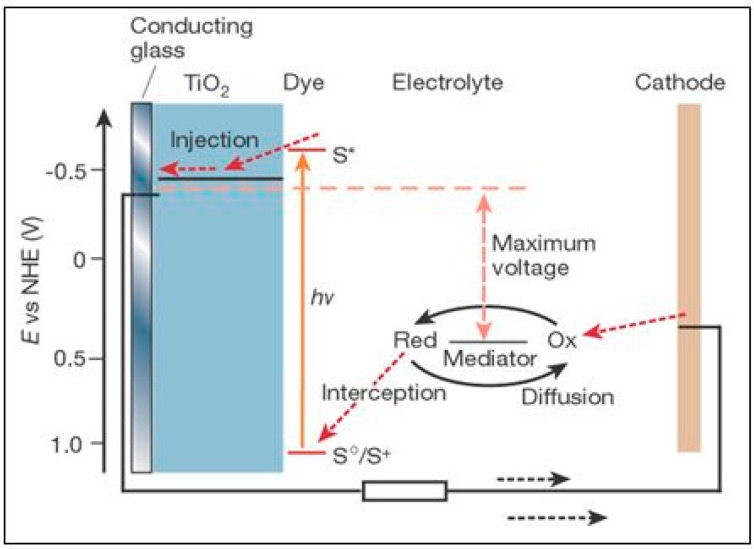
Working principle of dye-sensitized solar cell (DSSC) (reproduced from [76] with permission of Nature Publishing Group).

The greatest success has been achieved in the so-called mesoscopic Dye-Sensitized Solar Cell (DSSC), or Grätzel cell as named after its inventor [84]. The basic principle is illustrated in Figure 6. Upon photo-excitation of the chemically-anchored dye monolayer, electrons are injected to the conduction band of the mesoporous TiO_2_ substrate indirectly via an excited state (S*). Back electron transfer to the oxidized dye is prevented by rapid dye regeneration (reduction) by a donor species (I^−^) present at high concentration in the electrolyte. To complete the circuit, the oxidized form of the mediator (I_3_^−^) is discharged by electron flow through the external circuit to the Pt cathode. The energy difference between the TiO_2_ conduction band and the redox level of the mediator (I^−^/I_3_^−^) determines the maximum open circuit voltage (V_oc_ ≈ 0.8 V), while the IPCE generally exceeds 70% up to 700 nm, leading to photocurrent densities ≈ 15 mA·cm^−2^. With a typical “fill factor” (non-ideality in the power curve) of around 0.75, the resulting overall efficiency is ~10%. 

**Figure 7:**
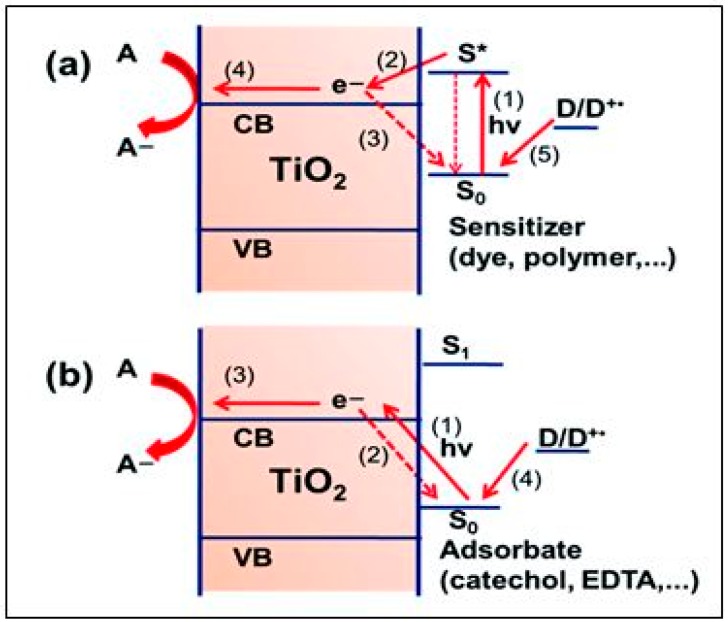
Scheme of charge transfer modes from sensitizer (S) to the TiO_2_ conduction band (CB): (**a**) indirect (type I); (**b**) direct (type II or LMCT) (reproduced from [95] with permission of the Royal Society of Chemistry).

Most recently, this has been extended to 13% while at the same time reducing costs, substituting the previous Ru-based panchromatic (black) dyes with more absorptive (Zn-based) donor-π-acceptor porphyrins and the slightly corrosive I^−^/I_3_^−^ redox mediator by Co^2+^/Co^3+^-based electrolytes [85]. Although the DSSC is strictly speaking a photovoltaic (PV) device rivaling conventional Si, their recent coupling into water splitting PEC cells has led to remarkably high efficiencies of H_2_ generation (*vide infra*) [61,62]. Future advances can be expected from co-sensitization utilizing mixed dye systems, one of which absorbs in the near infrared region [86,87,88,89,90,91]. Alternatively, by varying its surface binding configuration, a single dye may achieve charge injection by both *direct* (type II—as exemplified by catechol, EDTA, *etc.* (see Figure 7b [92]) and *indirect* (type I—see Figure 7a) modes, thereby rendering it more panchromatic in response [93,94]. Although it has not yet come to fruition, pure type II sensitization should increase DSSC efficiency by eliminating the electron injection overpotential, *i.e.*, the energy loss due to thermalization from the excited state of the dye (S*) in the conventional (type I) process [93]. The general prospects for exploiting direct ligand-to-metal (Ti^4+^) charge transfer (LMCT) absorption in photocatalysis over TiO_2_ have been reviewed [95]. It can be recognized by the appearance of a new absorption band absent in either of the free components. One serendipitous example is self-activation of hydrogen peroxide (which forms the yellow peroxotitanate complex on adsorption) for visible-driven environmental applications of TiO_2_ [95,96]. The most exciting recent discovery in the DSSC field is the advantage of utilizing panchromatic semiconductor alkyl-ammonium lead (tin) trihalide perovskites as solid-state (layer) sensitizers [97,98,99,100], simultaneously replacing both the conventional dye and redox mediator. This technology-disruptive advance should ease fabrication costs and accelerate the development of a cheaper alternative solid-state PV cell of similar durability and efficiency to Si.

In particulate systems, bulk doping of TiO_2_ with inorganic (metallic or non-metallic) elements has been a major strategy for visible sensitization in the last decade. Although this approach offers better long-term photostability (as compared to sensitization by organic dyes), the complexities of the solid-state chemistry have been decidedly more challenging. This is linked primarily to restricted solubility of dopants when introduced individually (1–2 at %), leading to insufficient visible light absorption. Furthermore, the concomitant introduction of defects that act as recombination centres has often led to efficiency losses. These are commonly O-vacancies but the dopant site itself can act deleteriously if present above a certain threshold concentration [101]. It is inadequate merely to impart colour to TiO_2_, e.g., from transition metal ions (TMI) with a d^1^–d^9^ electronic configuration. Such optical transitions, being mostly localized (d ↔ d) type, do not involve charge transfer and merely act as parasitic absorbers competing with genuine (delocalized) charge injection. In a few cases, intervalence charge transfer may be effective provided the energy state of the photo-reduced acceptor lies above the bottom of the conduction band, e.g., Ti^3+^, V^2+^, or Fe^2+^ [102]. Early interest in TMI doping in TiO_2_ was intended to improve the efficiency of charge separation and/or to extend the lifetime of surface-trapped carriers for photochemical action [103,104]. Any advantage to be gained by applying the TMI strategy to visible-light sensitization remains debatable as attested by more recent literature [105,106]. Indeed, it is increasingly recognized that incorporation of TMIs with empty (d^0^) or filled (d^10^) d-shells gets better results [107,108]. The recent flurry of excitement over the discovery of “black” TiO_2_, obtained by high-pressure hydrogenation [109], is fading since it was confirmed that little or no visible photoactivity is generated [110], even though UV activity can be dramatically increased. Nevertheless, it has led to theoretical modeling [111] and renewed interest in defect engineering in pure TiO_2_ [82,101,112].

**Figure 8:**
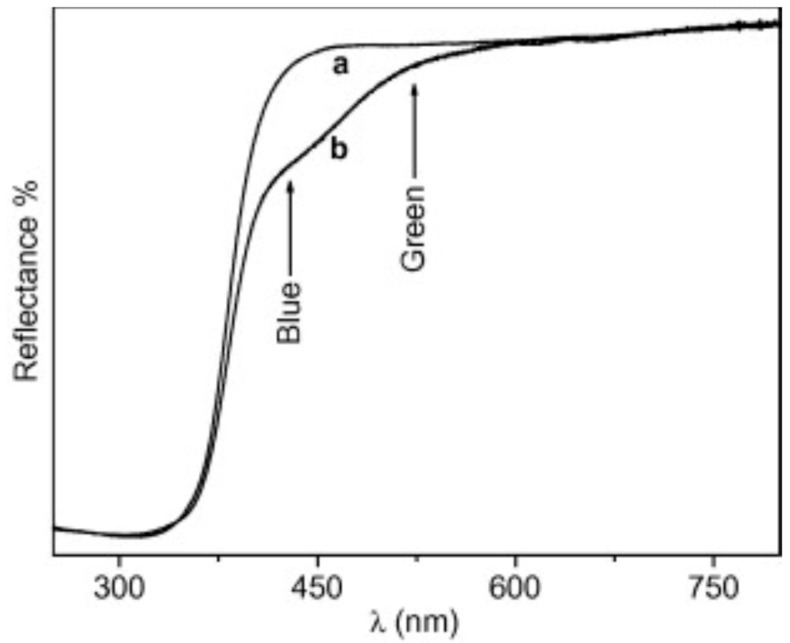
UV-visible reflectance spectra of (a) pristine TiO_2_ (b) N-doped TiO_2_ (reproduced from [126] with permission of Elsevier).

The advent of what are now considered “2nd generation” photocatalysts [82,113] was triggered by independent reports in 2001 and 2002 that doping with electron acceptors, *i.e.*, elements forming anionic species such as N [114] and C [115], was the most effective way to impart visible-light sensitization. This was soon corroborated [116,117,118,119], and studies were extended to include F [120], S [121], and P [122]. Awareness of the benefits (synergies) of anionic *co-doping* gradually followed [123,124,125]. Thanks to good underpinning by DFT (calculational) modeling [117,126,127], most studies have focused on modified N-TiO_2_ [128,129,130]. However, the visible sensitization effect is limited. N-TiO_2_ generally appears pale yellow due to the low level of dopant achievable (<2 at % N), conferring only weak absorption in the blue-green region (see Figure 8).

**Figure 9:**
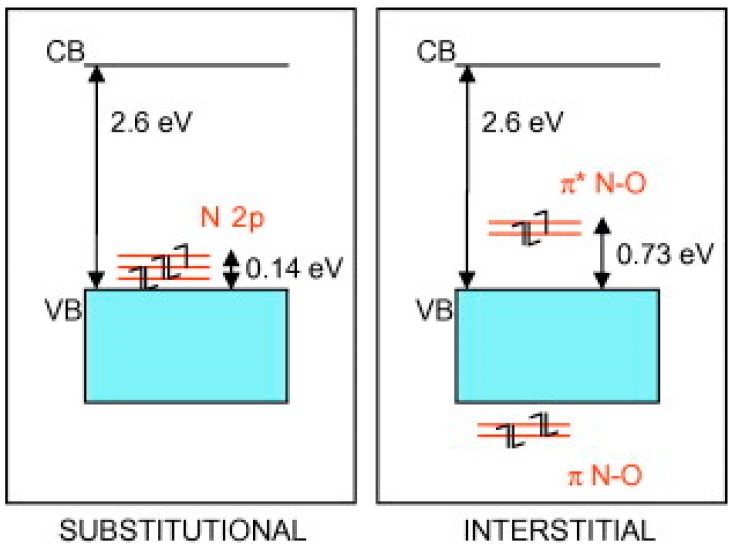
New energy states introduced into TiO_2_ by substitutional and interstitial nitrogen. (reproduced from [126] with permission of Elsevier).

Furthermore, a number of studies have found a loss in oxidative power upon illumination within this visible band, and have attributed this mainly to faster charge carrier recombination [131,132,133]. Di Valentin *et al.* [126] have shown that the most stable doping configuration depends on the chemical potential of ambient oxygen during preparation. Since the most common reagent, NH_3_, has reducing properties O-poor conditions prevail, favouring combination of two substitutional nitrogen sites compensated by one O-vacancy (2N_s_ + V_o_). The implied diamagnetic material of formula TiO_(2-3x)_N_2x_ has been affirmed as most likely by more recent modeling [127]. In an O-rich environment, a species consisting of an interstitial nitrogen atom associated with O (N_i_-O) may also be stable, interacting with lattice Ti atoms through its π-bonding states, as shown in Figure 9. In either case, new (N_2p_) energy states predicted to lie slightly above the valence band could be responsible for the observed visible absorption band around 450 nm. EPR spectroscopy has shown that irradiation within this band transfers an electron from the bulk diamagnetic N centre to the TiO_2_ surface leading to formation of superoxide species (O_2_**∙**^−^), a key activation process in environmental photocatalysis.

The current theoretical viewpoint on bulk doping is to take a donor/acceptor cooperative approach. This can be electro-neutral, e.g., “B + N” [134] or “Mo + 2N” [135], combinations that form intra-gap states extending from the bottom of the conduction band and top of the valence band, respectively. Charge compensation reduces the risk of introducing detrimental structural defects (interstitials, vacancies, *etc.*), while enabling the incorporation of higher dopant levels. Alternatively, even non-compensated p,n- type co-doping has been proposed. Using an excess of donor, e.g., Cr > N, the creation of states of intermediate energy results in a quasi-continuum visible absorption and an apparent bandgap energy of 1.5 eV [136]. The extrinsic states responsible for enhanced visible photoresponse often involve paramagnetic centres that can be explored by EPR [136,137].

One caveat on bulk doping should be mentioned. While re-affirming that N-TiO_2_ has visible activity in formic acid mineralization, the same study claimed IR spectroscopic evidence for defective Ti≡N bonds and correlated this with weaker UV photoactivity due to related loss of crystallinity [138]. It is not clear if this trade-off in performance is inevitable [130]. Finally, it should be recognized that the “band narrowing” strategy vis-à-vis solar fuel generation may ultimately be constrained by high overpotentials associated with key redox processes, e.g., water oxidation (*vide infra*). The corollary is that *co-catalysts* will have a more vital role to play in lowering kinetic barriers in visible-active semiconductors, materials of intrinsically lower redox power than pristine TiO_2_ (see Section 3.3).

**Figure 10:**
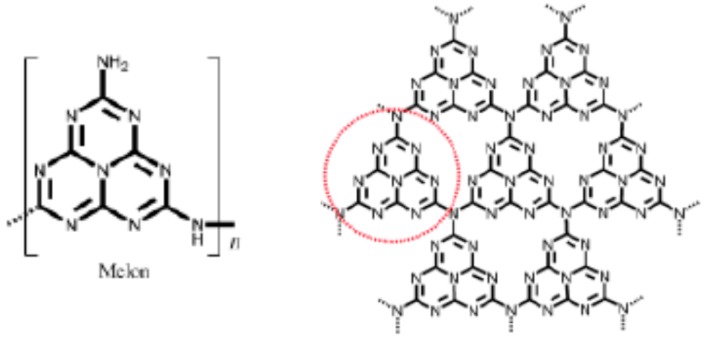
Melon and related tri-*s*-triazine unit (ringed) as building block for g-C_3_N_4_ (adapted from [143] with permission of Wiley).

**Figure 11:**
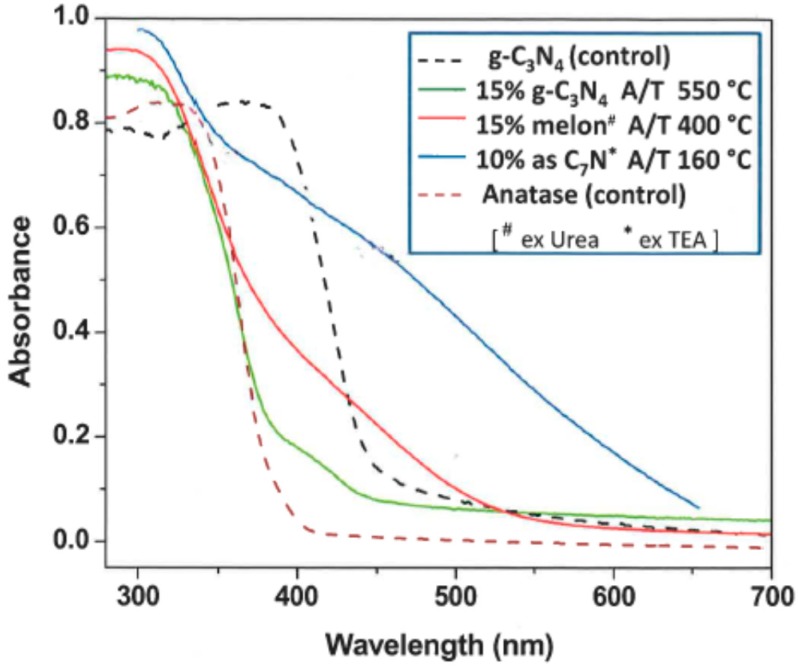
UV-Vis spectra of C/N-based sensitizers on biphasic anatase/titanates (adapted from [96,142] with permission).

A new class of more intensely coloured “multilayer-sensitized” titanias related to N-doped TiO_2_ has emerged recently. These are obtained via mild preparative routes like sol-gel, hydrothermal, *etc.*, where the N-source is usually organic instead of ammonia, the preferred reagent for bulk doping. Starting from urea, the organic moiety transforms stepwise during calcination into the yellow-brown melon structure based on tri-*s*-triazine (heptazine) rings [139,140,141], a process catalyzed by acidic (H)-titanates [142]. Insofar as melon is structurally related to the more-condensed (fully dehydrogenated) graphitic carbon nitride, g-C_3_N_4_, a visible-absorbing semiconductor *per se* [143], these N-modified materials resemble nanocomposites. Figure 10 shows examples of these structural tectons (building blocks) and their inter-relationship. Starting from amines or alkyl-ammonium salts, the material appears more intensely coloured (brown) already below 200 °C due to a strong absorption tail extending across most of the visible region [96,144,145,146,147]. However, unlike the case of melon, the exact identity of the chromophore is uncertain and it is thermally labile. Calcination weakens both visible absorption and photoactivity [96,148]. Representative UV-Vis spectra of various C,N-based sensitizers loaded onto biphasic anatase/titanates (A/T) are shown in Figure 11.

**Figure 12:**
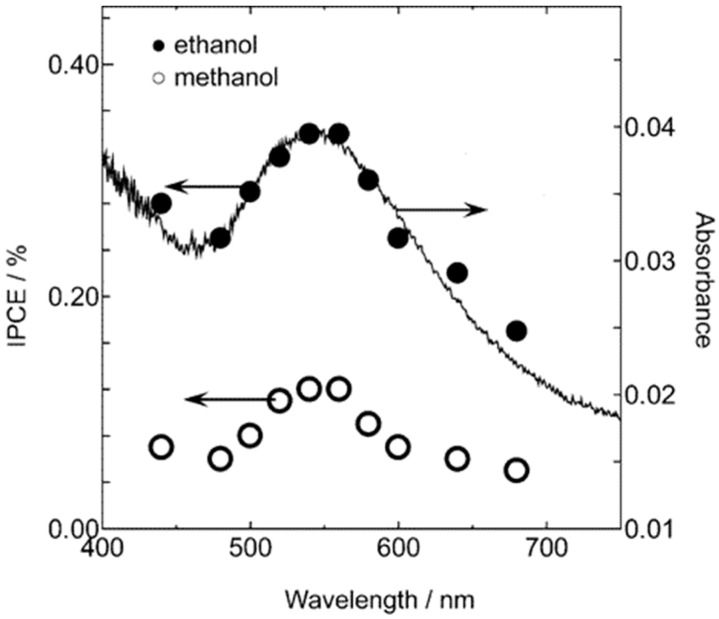
IPCE action spectrum in ethanol (methanol) photo-oxidation *vs.* LSPR (visible absorption) spectrum of gold in Au/TiO_2_ (adapted from [149]; copyright (2005) American Chemical Society).

Visible sensitization of TiO_2_ via “plasmonics” is another rapidly intensifying field that may yield 3rd generation photoactive materials [73,74]. Localized Surface Plasmon Resonance (LSPR) is responsible for the now familiar intense coloration of mono-dispersed colloidal noble metals like Au and Ag, in which the absorption band may be “tuned” by varying the particle size and shape. The first convincing report that visible-light-induced metal-to-semiconductor electron transfer can be induced in Au/TiO_2_ appeared in 2005 [149]. The action spectrum (IPCE) for photo-oxidation of ethanol was found to match the Au optical absorption peaking at ~550 nm (see Figure 12). 

**Figure 13:**
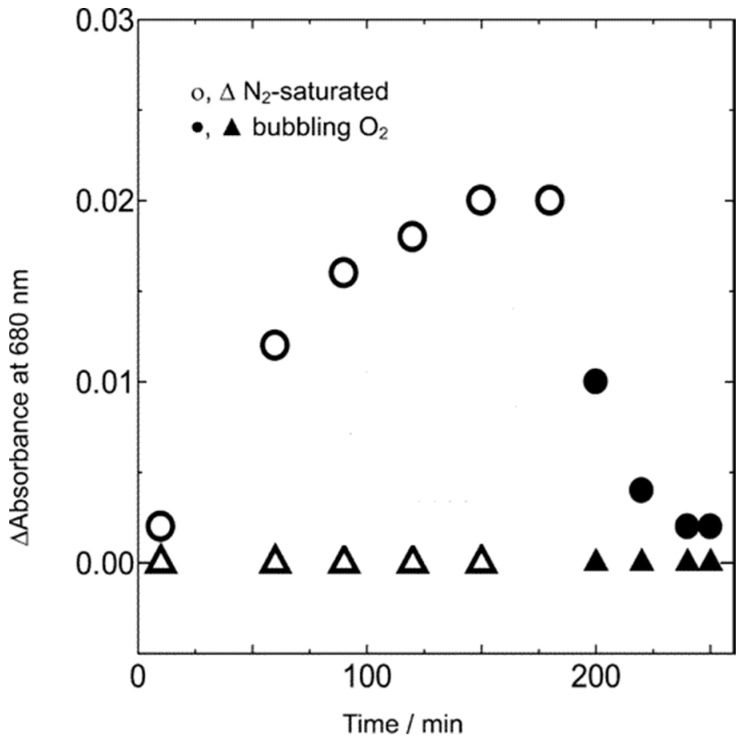
“Electron” absorption in TiO_2_ @ 680 nm induced by visible illumination (λ > 500 nm) of Au deposits and its quenching by ambient O_2_ (adapted from [149]; copyright (2005) American Chemical Society).

In addition, the characteristic spectrum of self-trapped electrons in TiO_2_ (λ_max_ ≈ 680 nm) developed under N_2_. As shown in Figure 13, this was quenched in the presence of O_2_ as electron acceptor. More extensive studies on Au/TiO_2_ and Ag/TiO_2_ have reached similar conclusions but efficiencies still need improvement [150,151,152,153,154]. The theoretical basis of plasmonics is developing rapidly, but two distinct types of interaction have already been identified [155,156,157,158,159]. The first is “hot electron” transfer from the metal to the semiconductor, which is the LSPR-induced analogue of indirect charge injection from a sensitizer dye in the DSSC, as described above. A second mechanism is also operative if their respective absorption bands overlap. This is termed plasmon resonant energy transfer (PRET), but its directionality may be reversed under UV irradiation (semiconductor → metal) via Forster resonant energy transfer (FRET). These “near-field” effects do not even require electrical contact at the interface. Notable examples are long-lived hot electron injection from Au to TiO_2_ [160], PRET from insulated Ag nanocubes to N-doped TiO_2_ [161]; or PRET from Au to α-Fe_2_O_3_ nanoplatelets [162]. Despite the promise of plasmonic sensitization, the long term economic outlook dictates a shift towards more earth abundant (but inevitably less stable) elements such as Cu [163,164,165], Al [166] and doped oxides [167].

### 3.2. Individual Alternatives to TiO_2_

Recent reviews affirm that applied photocatalysis research is still largely (>80%) based on TiO_2_, albeit in increasingly sophisticated (modified) forms [168,169,170]. Being amenable to nano-architecturing [170], and providing multi-phase heterojunctions for improved charge separation [171,172], this benchmark material has close to ideal properties as a photocatalyst [173,174] excepting its poor solar light response. One overdue task in TiO_2_ research is a more quantitative evaluation of the importance of trapping states that do not lead to fast recombination but, on the contrary, extend charge carrier lifetime into the seconds or minutes time domain [175,176,177,178,179,180,181]. Recent modeling studies on photoexcited anatase show that the energetics (site stability—surface *vs.* bulk) favour surface-trapping of both the hole and the electron [182], with beneficial implications for surface redox chemistry.

The search for visible-light active semiconductors that also satisfy other key criteria for practical photocatalysis on a large scale is a difficult task. While various alternative semiconductors exist with suitable bandgap (E_g_ = 1.5–3.0 eV), most are inferior to TiO_2_ in other respects, e.g., in having lower majority carrier conductivity, shorter minority carrier diffusion length (faster recombination), a less positive valence band edge (lower oxidizing power), instability under illumination (photo-corrosion), toxicity and/or high cost [183]. For these reasons, emphasis is now shifting to the development of type II (staggered bandgap) composites or tandem arrangements that perform complementary functions, coupled by directional electron transfer at the common heterojunction to “close the photochemical circuit” (*vide infra*) [111,183].

One notable exception that has emerged recently is graphitic carbon nitride (g-C_3_N_4_), which has a similar bandgap to N-TiO_2_ (E_g_ ≈ 2.7 eV—see also Figure 11), suitable energetics (band edge positions) for water splitting [142], and can be doped (bandgap tuned) and nano-textured to promote efficient charge migration [184,185,186]. However, a recent modeling study has identified a major kinetic constraint (large overpotential) linked to oxidative dissociation of water [187], helping to rationalize why co-catalysts are urgently needed for O_2_ evolution [188]. In contrast, modeling studies have shown that N-doping of anatase TiO_2_ may actually promote water dissociation [189]. 

Elsewhere, research into prospects for hematite (α-Fe_2_O_3_) has undergone a strong revival in the last decade, mainly due to efforts by the EPFL (Lausanne, Switzerland) group [162,183,190,191]. Pristine hematite is a cheap and stable indirect n-type semiconductor that absorbs visible light up to ~600 nm (E_g_ = 1.9–2.2 eV), offering a maximum photocurrent density of 12.6 mA cm^−2^, or a solar-to-hydrogen (STH) conversion efficiency (η_STH_) of ~16%. One limitation is its relatively low majority carrier (electron) conductivity, but this is readily overcome by incorporation of suitable dopants, e.g., Ti^4+^, Sn^4+^, Nb^5+^, *etc.* [192]. However, it also suffers from several more challenging (deleterious) properties as a photo(electro)catalyst. Having a conduction band edge too low in energy for proton reduction and a large overpotential for O_2_ evolution means water splitting over α-Fe_2_O_3_ will only work under external bias [183,191,193]. Furthermore, its high optical absorption depth (~400 nm [194]), coupled with a very short minority carrier (hole) diffusion length (~4 nm [195]) translates into a very low quantum efficiency for charge collection (IPCE). Nevertheless, structuring highly-crystalline deposits on the 20–30 nm scale has already raised the IPCE to over 30% with photocurrent densities exceeding 3 mA·cm^−2^ [190,191,196,197]. Reinforcing the contention that long-lived charge carriers are of key importance (*vide ultra*), application of transient optical and electrochemical techniques on α-Fe_2_O_3_ photo-electrodes held under positive (anodic) bias has shown a quantitative correlation between accumulated surface-trapped holes and photocurrent (electron) density [198]. The hole lifetime (τ = 0.1–1 s) is sufficient for photo-oxidation of water, which has a rate constant in the range 0.1–10 s^−1^ [199,200]. The holes are reported to be of two distinct types, O_2p_ (O^−^) and Fe_3d_ [Fe^n+^ (*n* > 3)], but both have similar activity [201].

### 3.3. Tandem (D4) Photoelectrochemical Cells, Composites, and the Role of Co-catalysts in Water Splitting 

It is 30 years ago now that visible light-driven electron transfer from CdS to TiO_2_ in an aqueous suspension of aggregated nanoparticles was first demonstrated [202]. The STH efficiency for the composite was very low but better than either of the pure components due to effective spatial separation and localization of electrons into TiO_2_ (for proton reduction), the holes remaining on CdS (for H_2_S oxidation) due to the relative energetics of the respective band edges (type II—see Figure 5 [82]). Here, the particles act as complementary self-biasing “photoelectrochemical diodes” in an efficient S2 mechanism (2 photons per H_2_ molecule). Since that time, remarkable progress has been made in the visible-driven reduction half-reaction of water splitting:

2 H^+^ + 2 e^−^ → H_2_ [E° (V) = 0.00 − (0.059 × pH)] (5)

**Figure 14:**
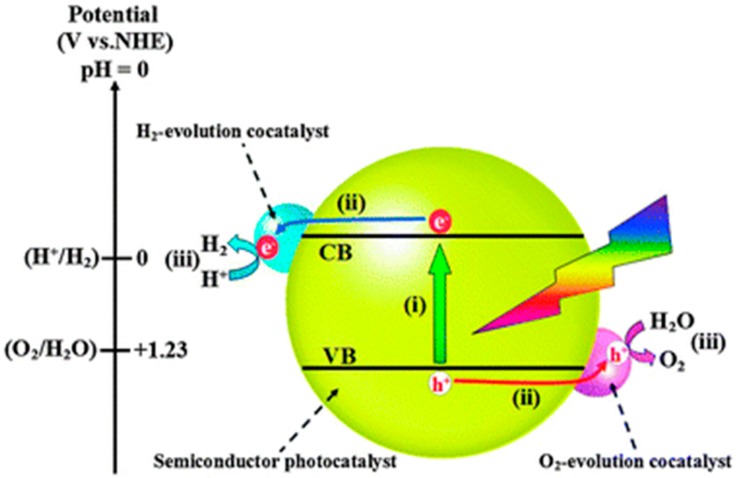
Photocatalytic water splitting over a visible-absorbing semiconductor loaded with H_2_- & O_2_-evolution co-catalysts (reproduced from [209] with permission of the Royal Society of Chemistry).

**Figure 15:**
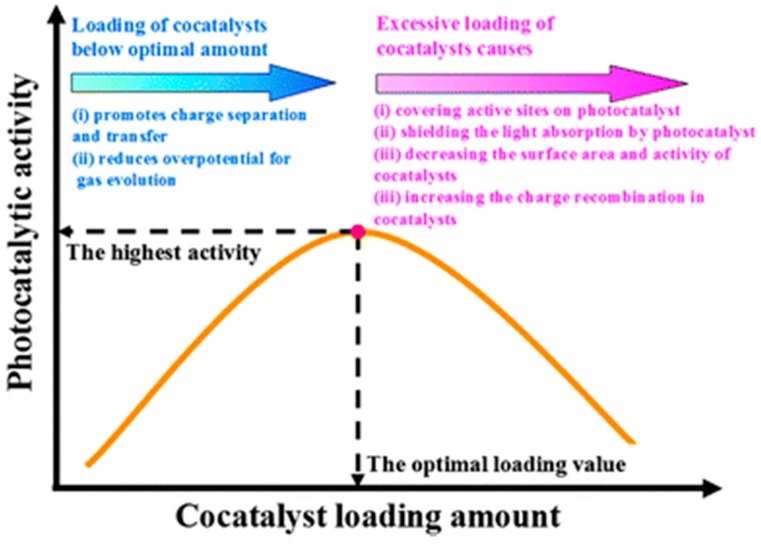
Principle of optimum loading of cocatalyst on a visible-absorbing semiconductor photocatalyst (reproduced from [209] with permission of the Royal Society of Chemistry).

A quantum yield of 93% was recorded over Pt/PdS/CdS at 420 nm, evolving 9 mmol/h H_2_ with sulphide/sulfite as sacrificial donors [203]. Conventional wisdom has it that the low levels of Pt (0.3%) and PdS (0.13%) act as cocatalysts [51,53,203,204]. PdS is believed to promote oxidation of S^2−^ and SO_3_^2−^ and transfer electrons to CdS. However, recent literature suggests that the combination of PdS and CdS may also be classified as an optical tandem system. PdS is an n-type semiconductor with a bandgap of ~1.6 eV and under investigation as a photovoltaic material *per se* [205,206]. It confers extra absorption in CdS composites that extends into the near IR region [207,208]. Unfortunately, the rarity of Pt and Pd, the tendency of sulphides to photocorrode, and the toxicity of cadmium ion, all militate against their use on a large scale. As shown in Figure 14, Pt as cocatalyst traps electrons from the semi- conductor to discharge protons, forming Pt-H bonds of ideal (intermediate) strength for H-H combination and desorption as molecular H_2_ (Sabatier Principle). While often black in appearance, co-catalysts are strictly not photoactive and promote only dark elementary steps in the reaction. As shown in Figure 15, there is an optimum amount due to competing (beneficial and deleterious) effects. In practice loadings fall below 1%, fortuitously mitigating costs (many are precious metal-based, e.g., IrO_2_, RuO_2_, *etc.*) while minimizing parasitic light absorption. Prospects for alternative earth-abundant cocatalysts in photocatalytic water splitting have been reviewed [209]. Promising substitutes for Pt in H_2_ evolution under neutral or alkaline conditions are Ni nanoclusters [210], Ni/Mo alloy [211,212], and Cu(OH)_2_ [213].

The water oxidation half reaction [or oxygen evolution reaction (OER)]:

2 H_2_O → O_2_ + 4 H^+^ + 4 e^−^ [E° (V) = +1.23 − (0.059 × pH)] (6)

is the main obstacle to efficient water splitting as it suffers from a large activation energy barrier (overpotential > 0.4V) due to the necessary transfer of 4 charges per O_2_ molecule in a complex proton-coupled electron transfer mechanism [46,214]. Nature’s catalyst in photosynthetic water oxidation is the CaMn_4_O_x_ cubane-related molecular complex [215]. Traditionally, simple though expensive oxides like RuO_2_ and IrO_2_ have been used at the anode of PEM (acid) water electrolyzers [216], while mixed oxide Ni and Co spinels or perovskites (with inclusion of Cu) are favoured in alkali electrolyzers [217]. In heterogeneous particulate systems, the earth-abundant oxide CoO_x_ loaded onto LaTiO_2_N had an OER quantum efficiency (Φ_O2_) of 27% at 440 nm [218], while CoO_x_ or MnO_x_ on TiO_2_ nanosheets achieved Φ_O2_ ≈ 15% at 365 nm [219]. In overall water splitting, only composite (tandem) absorbers, each optimized (with cocatalysts) for a single half-cell reaction have achieved quantum efficiencies greater than 5% (*vide infra*). Most progress has been made with oxysulphides or oxynitrides of d° or d^1^° metal cations [107,220]. It should also be noted that quantum efficiency (Φ) values are not to be mistaken for η_STH_. The last is theoretically ~10% for an absorber with a 500 nm absorption cut-off even at 100% quantum efficiency (Φ = 1). In reality, despite intensive efforts over the last three decades η_STH_ values for particulate systems have yet to exceed 1% [220,221].

**Figure 16:**
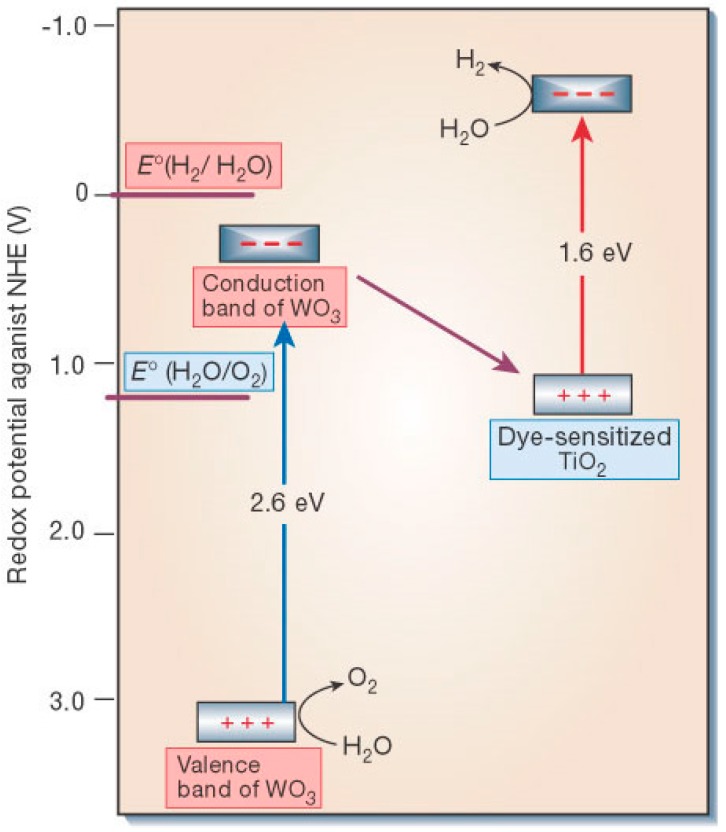
Example of Z-scheme (4D): WO_3_ photoanode (E_cb_ < E°_H^+^/H_2__) coupled to dye-sensitized TiO_2_ (reproduced from [76] with permission of the Nature Publishing Group).

**Figure 17:**
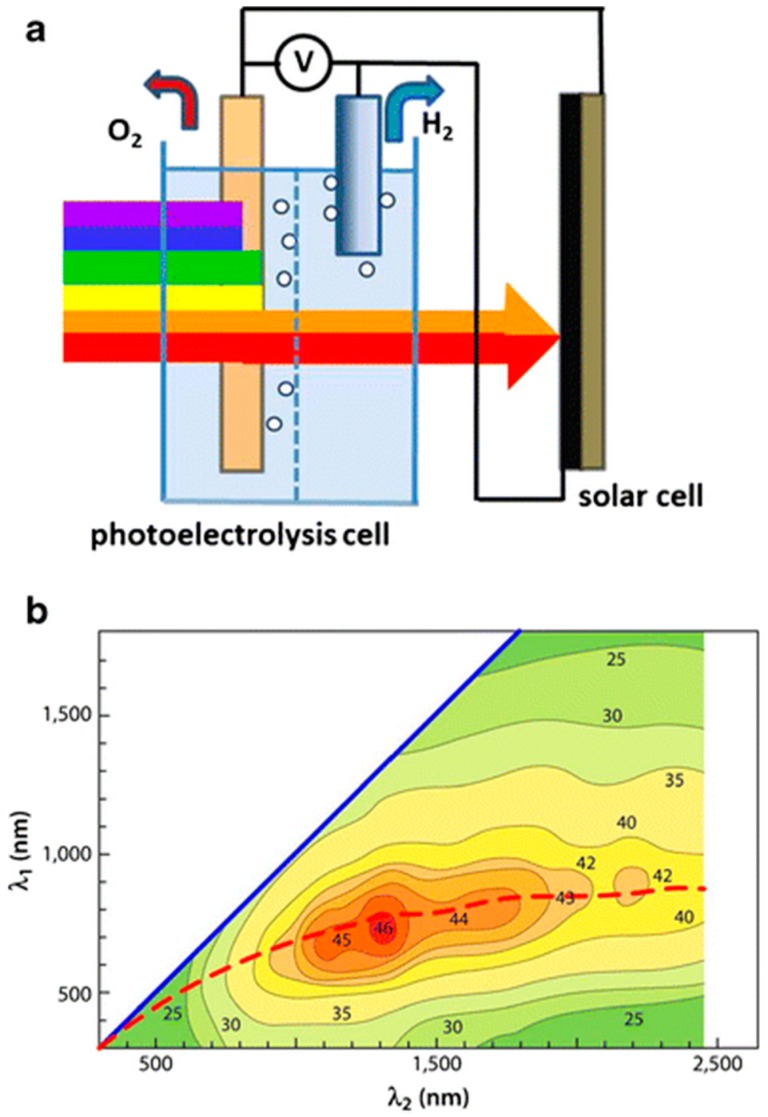
(**a**) Water splitting tandem cell: photoanode passes NIR light to solar cell, giving cathodic bias for H_2_ evolution; (**b**) Dual absorber efficiency curve (η_STH_ > 40% at λ_a_ ≤ 750nm (1.6 eV), λ_sc_ = 750–1300 nm (0.95 eV) (reproduced from [200] with permission from Springer).

Prospects for PEC cells look promising due to the efficient separation, collection and transport of photo-separated charges in a wired system. For most photoanodes, e.g., α-Fe_2_O_3_, WO_3_, or other materials of more suitable bandgap (E_g_ = 1.4–2.0 eV), the conduction band energy is so positioned that any injected electrons can only thermalize into the valence band of the photocathode, e.g., TiO_2_, where they effectively neutralize holes created by direct photo-excitation (of TiO_2_). By analogy with photosynthesis, such a configuration is generally referred to as a Z-scheme as originally proposed for spatially separated photoelectrodes [75]. Since most electrons reaching the photocathode conduction band are from the photoanode sensitizer and undergo two successive excitation steps, the mechanism is said to be of type D4 (4 photons per H_2_), As shown in Figure 16 for a visible-absorbing WO_3_ photoanode, if bare TiO_2_ is replaced by a photoactive cathode (or a solar cell whose cathode is configured to evolve H_2_) the theoretical combined efficiency can rise substantially due to wider light harvesting. This is most notably so (η_STH_ > 40%) in a series arrangement with an “in- front” photoanode (E_g_ ~1.8 eV) that absorbs visible wavelengths to evolve O_2_ from water. The transmitted near-IR light is incident on the solar cell (E_g_ ~0.95 eV), which provides a voltage bias for H_2_ evolution at the cathode (see Figure 17). The Z scheme principle has been extended to particulate systems but with limited success [50,107]. An added complexity here is the need to promote interparticle electron transfer using a redox mediator, e.g., IO_3_^−^/I^−^, in solution. However, this suffers from “chemical short-circuiting”, *i.e.*, competitive reactions between water and the mediator, and especially reaction of its oxidized form, necessarily present in excess, with product H_2_. Nevertheless, Maeda *et al.* [222] have reported a respectable overall quantum efficiency of 6.3% at 420 nm for a Pt-doped ZrO_2_-protected TaON “cathode” (for H_2_ evolution) suspended with PtO_x_-loaded WO_3_ as “anode” (for O_2_ evolution). Better prospects may lie in elimination of mediators and the development of “all-solid-state” Z-scheme analogues, *i.e.*, composite particles with heterojunctions [223,224,225].

**Figure 18:**
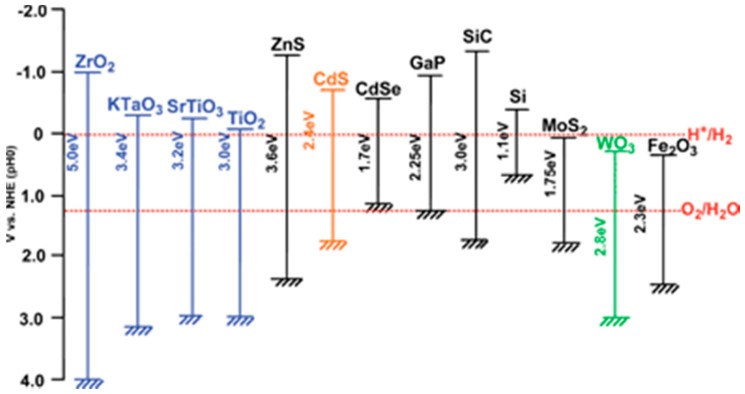
Bandgaps and band edge positions of representative semiconductors in relation to the redox potentials for water splitting at pH = 0. (Reproduced from [220] with permission of the Royal Society of Chemistry).

The choice of materials for practical tandem PEC cells is restricted. Figure 18 shows the bandgap and bend edge position of representative semiconductors. They must be cheap (earth-abundant) and stable ideally in strongly acidic and/or alkaline conditions for good electrolyte conductance. 

Bandgaps must also be higher to compensate for inevitable voltage losses, e.g., device series (ohmic) resistance, and electrode overpotentials. Nevertheless, the efficiencies of these tandem devices are expected to exceed 25% [226,227]. Major advances towards this goal were reported by Nocera *et al.* (η_STH_ = 4.7%, 2.5% wireless) [62] and Grätzel et al (η_STH_ = 3.1%) [61], but with a quite different system approach. Nocera’s design consisted of a triple-junction (T6 or 6-photon) amorphous Si absorber loaded with cobalt phosphate (for the oxygen evolution reaction—OER) [228]) and Ni/Mo alloy (for H_2_ evolution [211,212]) on a Ni mesh in phosphate or borate/nitrate buffer. The wireless layout suffered from an added resistance loss due to the longer migratory path imposed on proton transport from the front (anode) to the rear (cathode) of the cell. Grätzel’s design was based on a single WO_3_ (Fe_2_O_3_) photoanode coupled to a single DSSC, as shown in Figure 16 and Figure 17. It is not clear if only Fe_2_O_3_ or both were loaded with IrO_2_ co-catalyst. Continuing their exploration of a buried junction PV configuration with loaded electrocatalysts (EC), in which the Si absorber is protected from the electrolyte, the Nocera team most recently achieved η_STH_= 10% with these so-called PV-EC tandem devices [78]. It consists of 4 single-junction crystalline Si solar cells connected in series to a NiMo cathode and a nickel borate anode, all immersed in a borate buffer (pH 9.2). The Ni-based anode is comparable in performance and cheaper than cobalt phosphate, but needs prior anodization (after deposition) to create the mixed-oxidation Ni^III/IV^ state responsible for OE activity [229]. However, both Ni and Co salts can be electrodeposited, conveniently forming the anode *in-situ* from divalent ions in the appropriate buffer. The latest efficiency advance reported by the Grätzel team is η_STH_ = 12.3% in an analogous PV-EC device [77]. This was achieved with two DSSCs connected in series, each providing a short-circuit photocurrent density of 21.3 mA·cm^−2^, open-circuit voltage (V_oc_) = 1.06 V, and a fill factor of 0.76. A combined solar-to-electric power conversion efficiency of 15.7% was attained with superior light harvesting by lead iodide perovskite (CH_3_NH_3_PbI_3_) sensitizers, prepared using a simple two-step spin-coating method at 100 °C. A cheap Ni-foam supported Ni/Fe layered double hydroxide, obtained by one-step hydrothermal growth, served as both cathode and anode in 1M NaOH electrolyte. It should be noted that a comparable performance may be obtained in PV-driven (“brute force”) electrolysis, *i.e.*, by combining state-of-the-art PV modules and electrolyzers optimized independently. For example, η_STH_ ≈ 12% has been reported in standalone systems where the voltage was maintained at ~1.7 V per cell (in a 20 cell PEM electrolyzer stack) with a DC-DC converter [230,231]. The PEC cell is a less expensive single integrated unit, provides a higher open circuit photovoltage, and reduces potential loss channels. However, it is susceptible to electrolyte resistance and polarization losses [227,232], especially under neutral conditions needed for operational stability of many earth-abundant co-catalysts. In view of such complications and exciting results with hybrid (PV-EC) devices, co-development of PV-electrolyzers and PEC water splitting cells may offer the best prospects [233,234].

## 4. Hydrogen Peroxide as Solar Fuel and Sustainable Chemical

Water splitting to H_2_ and O_2_ has been considered the “Holy Grail” of chemists working in the energy field. However, the co-production of H_2_ and H_2_O_2_ is arguably a yet more valuable process:

2 H_2_O → H_2_ + H_2_O_2_ [∆G° = +342 kJ/mol H_2_O_2_] (7)

and may actually be easier, *i.e.*, the kinetic barrier may be lower, because it is just a 2 e^−^ process:

2 H_2_O → 2 H^+^ + 2 e^−^ + H_2_O_2_ [E° = −1.77 V] (8)

This requires two photons at 171 kJ/mol (photons), corresponding to wavelengths below ~690 nm, which comprises ~50% of the solar power spectrum. Hydrogen peroxide is a valuable commodity chemical serving as a green oxidant in environmental clean-up, pulp bleaching, detergents, *etc.* [235]. It is now made largely by the *Anthraquinone Process* but research has intensified in recent years into direct catalytic synthesis from H_2_ and O_2_:

H_2_ + O_2_ → H_2_O_2_ (9)

which is exothermic (∆H° = −136 kJ/mol) and a competitive option for small-scale on-site production (< 10^4^ t/y) [236,237]. However, it deals with potentially explosive mixtures and only works efficiently over expensive rare metal (Pd or Pd/Au) catalysts. It is also an example of a highly selective partial oxidation reaction in which reaction with a second H_2_ molecule:

H_2_O_2_ + H_2_ → 2 H_2_O_(g)_ (10)

is even more exothermic (Equation (10) is the reverse of Equation (7)) and must be kinetically inhibited [238]. A PtHg_4_/C electrocatalyst was shown to be active and highly selective for H_2_O_2_ synthesis, as predicted by DFT modeling [239]. Alloying leaves an isolated surface Pt atom for hydroperoxide (HOO*) stabilization in the on-top position while eliminating the hollow adsorption sites preferred by activated oxygen (O*) species, thereby inhibiting water as product. Unfortunately, it is still a rare-metal based formulation. The cathodic half-cell reacton for peroxide synthesis from water (Equation (8)) can be written in two ways depending on the electron acceptor. 

For co-production of H_2_ (Equation (7) overall) this is:

2 H^+^ + 2 e^−^ → H_2_ [E° = 0.00 V] (11)

However, peroxide can also be synthesized by O_2_ reduction:

O_2_ + 2 H^+^ + 2 e^−^ → H_2_O_2_ [E° = 0.68 V] (12)

Summing the two half reactions (8) and (12), each yielding one peroxide molecule, gives Equation (13):

2 H_2_O + O_2_ → 2 H_2_O_2_ [E° = −1.09 V] (13)

This is still a potentially visible-driven endergonic process so that H_2_O_2_ alone can be considered as an energy carrier derivable from cheap reactants. Supplied commercially as 30% aqueous solution (~9M) it is already in an energy dense form, unlike H_2_ gas, and this underlines recent interest in peroxide as a solar fuel. Having acceptor and donor properties (reverse of Equations (8) and (12), respectively), the theoretical voltage of a “direct H_2_O_2_” fuel cell based on its own dismutation (reverse of Equation (13)) is 1.09 V. Although it enables a simplified fuel cell design (use of a single compartment is possible), the current densities are not yet of technical interest [240,241,242,243,244,245,246]. Alternatively, as a more powerful oxidant (than O_2_) supplied to the cathode, it increases the operating voltage in cells based on H_2_ [247] or liquid fuels such as aqueous NaBH_4_ [248,249] and ethanol [250]. However, its powerful oxidizing properties and susceptibility to decomposition by traces of metal (ions) and redox-active surfaces can also lead to under-performance (mixed potentials) and introduces more stringent material compatibility issues, especially concerning long-term stability of polymer membranes and possibly carbon as an electrocatalyst support. Potential cathode materials explored to date include PbSO_4_ [240], Au on Vulcan [249], and LaNiO_3_ on N-doped graphene, which promotes the ORR [251].

In energetic terms, the photosynthetic route involving co-generation of hydrogen (Equation (7)) would be preferred as long as the H_2_O_2_/H_2_ mixture remains stable. This has been reported for platinized calcium niobate [252] and Pt/TiO_2_ [253]. Both showed relatively low quantum efficiencies (Φ_uv_ < 1%) but the 1:1 product stoichometry of Equation (7) was confirmed. A liquid water environ-ment would probably impede further reaction because any H_2_O_2_ dissolves selectively. However, little peroxide was found in solution, most remaining associated with the catalyst in a form seemingly immune to any back-reaction with H_2_. This is consistent with previous literature showing that, in the absence of O_2_, a stable form of peroxide (O_2_^2−^, HO_2_^−^, *etc.*) builds up and deactivates the photo-process in a few hours, typically affording only micromoles of products. Although the intermediate(s) can be decomposed easily to yield O_2_ (the “missing” product in early studies of water splitting [254,255]), a way must be found to displace it as intact H_2_O_2_, e.g., by exploring more weakly-adsorbing (non-oxide) semiconductors and/or loading an oxidation co-catalyst. A viable system needs to sustain *milli*moles per hour productivity indicative of a broad spectral response and quantum yields exceeding 10%. This may be achievable but not easily recognized in practice because the peroxide, once formed, can decompose adventitiously, e.g., in the presence of trace metal ions, or excited by the UV component of a solar simulator, *etc.* Such H_2_O_2_ production rates (Φ_uv_ = 1%–30%) have been reported over quantum-sized ZnO and TiO_2_ particles but only under oxygenated conditions, implying that photo-reduction of O_2_ (Equation (12)) is the main source of peroxide [256]. Fluorination of TiO_2_ improved dramatically the yield by weakening the surface adsorption of peroxide, a precursor step in self-decomposition [257]. 

**Figure 19:**
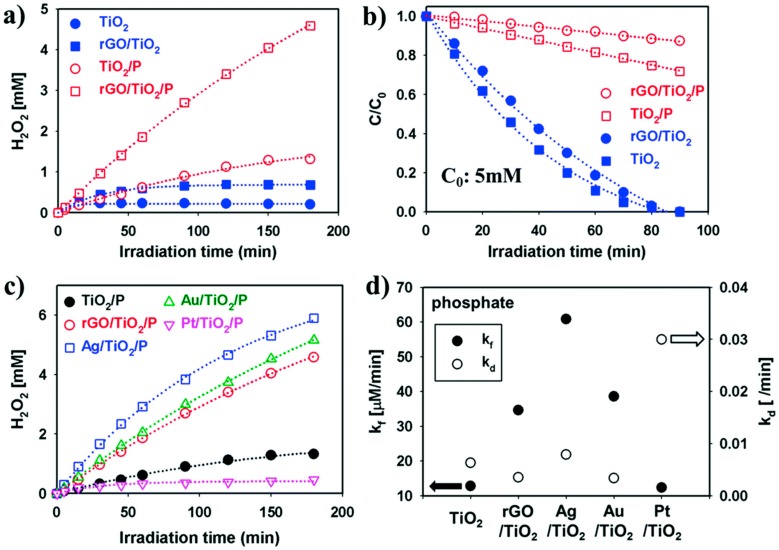
(**a**) Effect of pre-treating TiO_2_ with phosphate on H_2_O_2_ photosynthesis (λ > 320 nm), and (**b**) 5 mM H_2_O_2_ decomposition over 6 wt % rGO/TiO_2_ (0.5 g/L) in O_2_-saturated aqueous buffer (pH 3) containing 5 vol % 2-propanol as hole scavenger; (**c**) Comparison of H_2_O_2_ synthesis rates over rGO/TiO_2_(P) and 1 wt % metalized TiO_2_(P) samples; (**d**) Rate constants of H_2_O_2_ synthesis (k_f_) and decomposition (k_d_) over TiO_2_(P)-supported composites (reproduced from [258] with permission of the Royal Society of Chemistry).

**Figure 20:**
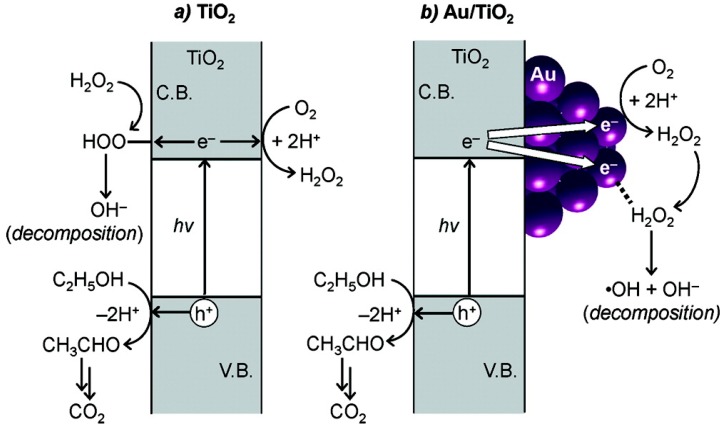
Scheme of synthesis and decomposition of H_2_O_2_ on (**a**) TiO_2_; and (**b**) Au/TiO_2_ photocatalysts. (Reproduced from [259]. Copyright (2012) American Chemical Society).

**Figure 21:**
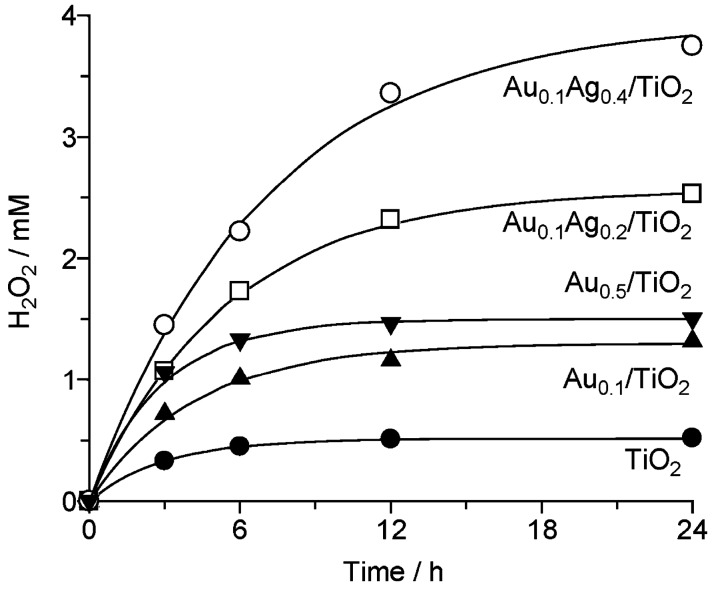
Build-up of photostationary levels of H_2_O_2_ on Au-Ag/TiO_2_ catalysts (5 mg) in 5 mL aerated 4% aqueous ethanol (λ > 280 nm, I_uv_ ≈ 14 mW)). (Reproduced from [259]. Copyright (2012) American Chemical Society).

As shown in Figure 19, the same effect was seen in a reduced graphene oxide (rGO)/TiO_2_ composite in which the TiO_2_ surface was phosphated to prevent simultaneous degradation, which is otherwise responsible for the attainment of photostationary product levels [258]. Alternatively, as shown in Figure 20 and Figure 21, alloying Au (on TiO_2_) with Ag suppresses selectively the intrinsic tendency of Au to simultaneously decompose its own product, thereby raising photostationary yields [259]. In contrast, it has been reported that TiO_2_ *per se* is a poor catalyst in H_2_O_2_ decomposition due to its low affinity for the OH**·** radical [260,261]. This powerful but non-selective oxidant:

HO^**·**^ + H^+^ + e^− ^ → H_2_O [E° = 2.80 V] (14)

is produced from hydrogen peroxide by an electron donor:

H_2_O_2_ + H^+^ + e^−^ → HO^**·**^ + H_2_O [E° = 0.72 V] (15)

uch as Fe^2+^ in the Photo-Fenton process, one of a variety of advanced oxidation processes (AOP) [13,96,262]. Unfortunately, it also promotes the autocatalytic decomposition of peroxide [263]:

H_2_O_2_ + OH^**·**^ → H_2_O + HO_2_^**·**^ [E° = 1.40 V] (16)

HO_2_^**·**^ → O_2_ + H^+^ + e^−^ [E° = −0.05 V] (17)

Hole scavengers (H**·** donors) always increase decomposition rates, suggesting that the 2 e^−^ water oxidation half-reaction (Equation (8)) is rate-determining in the synthesis. Under these conditions, *i.e.*, with little H_2_ or H_2_O_2_ produced from water, the process is of no interest as an energy conversion scheme. Nevertheless, the peroxide can still be considered a value-added green reagent obtained efficiently and cheaply by photo-oxidation of organic wastes. Evidence has just been reported for visible-driven H_2_O_2_ photosynthesis from oxygenated ethanol over pristine g-C_3_N_4_ [264], whose conduction band minimum (E° ≈ −1.3 V [265]) exceeds the reduction potential for the O_2_/O_2_^−^ couple (E° ≈ −0.3 V [266]). A 1,4-endoperoxide intermediate stabilized by the g-C_3_N_4_ surface was identified by Raman spectroscopy. Addition of Pt had a deleterious effect on yield due to its tendency to break the O–O bond [239].

## 5. Photoreforming of Bio-Oxygenates

In the renewable energy scheme under consideration (*cf.* Figure 1), bio-oxygenates like sugars, alcohols, and polyols, all serve as CO_2_-neutral energy carriers provided that their incipient H_2_ can be extracted efficiently by catalytic reforming with steam [31,32,55,56]:

C_n_H_m_O_k_ + (2n − k) H_2_O → n CO_2_ + (2n + m_/2_ − k) H_2_ (18)

Despite being highly endothermic, Equation (18) is favoured thermodynamically above a threshold temperature due to the large volume expansion (entropy factor). Input thermal energy is converted into chemical energy (H_2_) and represents a significant gain in exergy (20%–30%), as can be seen by comparing the heats of combustion of reactant and product. This is known as “chemical recuperation” [267,268], and pre-reforming of natural gas is likely to be incorporated into future gas turbine technologies [269]. Methanol is not currently made on a large scale from biomass or renewable H_2_ and can be readily reformed by conventional (thermal) catalysis [23,24,25,28]. In contrast, ethanol comprises 90% of biofuel production and, due to its high growth forecast [270], is now being considered as a renewable platform chemical, e.g., for butadiene synthesis [271]. Ethanol is also a good model oxygenate as it is one of the simplest compounds containing C–C, C–H, and C–O bonds. However, its catalytic conversion in high activity and selectivity poses a major challenge [272,273].

**Figure 22:**
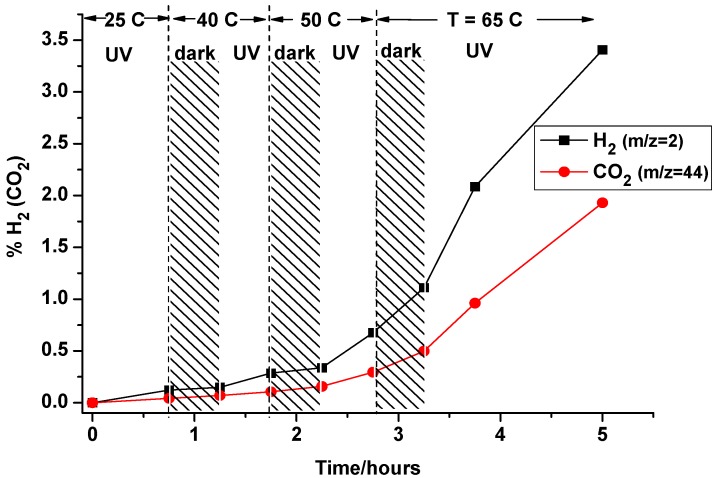
MS response (H_2_/CO_2_) showing effect of temperature on rate of vapour-phase CH_3_OH photoreforming over Pt/TiO_2_ (reproduced from [288] with permission of RSC).

**Figure 23:**
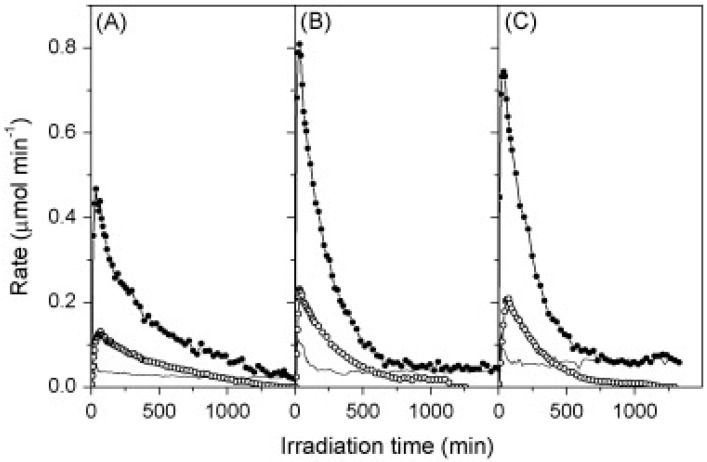
H_2_ (full circles) & CO_2_ (closed circles) evolution during liquid-phase (0.37 mM) aqueous glycerol reforming at (**A**) 40 °C; (**B**) 60 °C; (**C**) 80 °C (reproduced from [291] with permission of Elsevier).

Bio-ethanol obtained by fermentation of glucose:

C_6_H_12_O_6_ → 2 CO_2_ + 2 C_2_H_5_OH (19)

is an excellent energy carrier since almost the entire heating value of the original sugar (~2800 kJ/mol) is retained in the product (two moles liquid ethanol at 1365 kJ/mol). Ethanol steam reforming (ESR):

C_2_H_5_OH (g) + 3 H_2_O (g) → 2 CO_2_ + 6 H_2_ [∆H° = +174 kJ/mol] (20)

raises the fuel value substantially (six moles H_2_ at 286 kJ/mol). In addition, Equation (20) has a crossover (∆G° ≤ 0) temperature as low as 210 °C [19], suggesting it could be driven by “low-quality” heat provided a suitable catalyst can be found. This explains the major interest in ESR in recent years, e.g., as an on-board source of H_2_ for PEM fuel cell (electric) vehicular propulsion [274,275]. However, the low rates encountered over many oxide-supported transition metals (Pt, Ni, Co, Rh, Ru) necessitate working above 400 °C, where rapid deactivation by coking ensues, possibly linked to acetic acid intermediate [30,276,277,278]. DFT modeling supports experimental data showing that the rate-determining step in ESR is initial dehydrogenation to acetaldehyde [279]:

C_2_H_5_OH_liq_ → CH_3_CHO + H_2_ [∆H° = +85 kJ/mol] (21)

a modestly endothermic reaction but with an apparent activation energy (E_app_) as high as +150 kJ/mol [280,281]. This is typically followed by decarbonylation of the aldehyde [281,282]:

CH_3_CHO → CO + CH_4_ [∆H° = +7 kJ/mol] (22)

an almost thermoneutral process giving an undesirable alkane product. Photocatalysis may circumvent these activity/selectivity issues because it works by an alternate mechanism (lowering E_app_) and at low temperature where deleterious side reactions are inhibited. The pioneering work of Pichat *et al.* [283,284] established that photo-dehydrogenation (PDH) of alcohols (see Equation (21)) proceeds in high quantum efficiency (Φ_uv_ ≥ 0.1) over Pt/TiO_2_. More recently, photo-assisted water-gas shift (WGS) [285], photoreforming of methanol [286,287], and combined photo-/thermal reforming of methanol (or glycerol) to H_2_/CO_2_ co-products over Pt/TiO_2_ [288] (or Pd/TiO_2_ [289]) have been studied. As shown in Figure 22 and Figure 23, mild heating is a useful adjunct in photocatalysis when dark processes are rate-controlling. However, despite the sharp rise (×2–×5) in quantum efficiency (Φ_uv_ ≈ 7% for CH_3_OH at 65 °C [288]) and better reaction stability (due to more complete product recovery [288,290]), this synergism has still not been widely exploited. 

**Figure 24:**
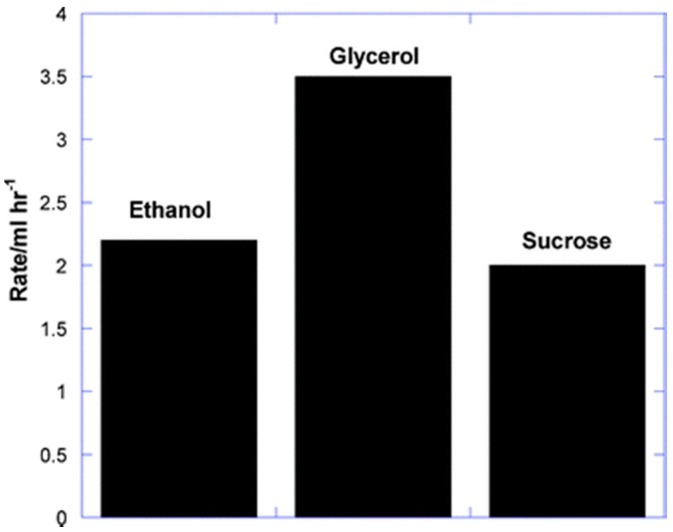
Rate of H_2_ evolution by photo reforming of 3 important bio-oxygenates (1% in H_2_O) over 0.3 wt % Pd/TiO_2_ (P25) (reproduced from [298] with permission of RSC).

**Figure 25:**
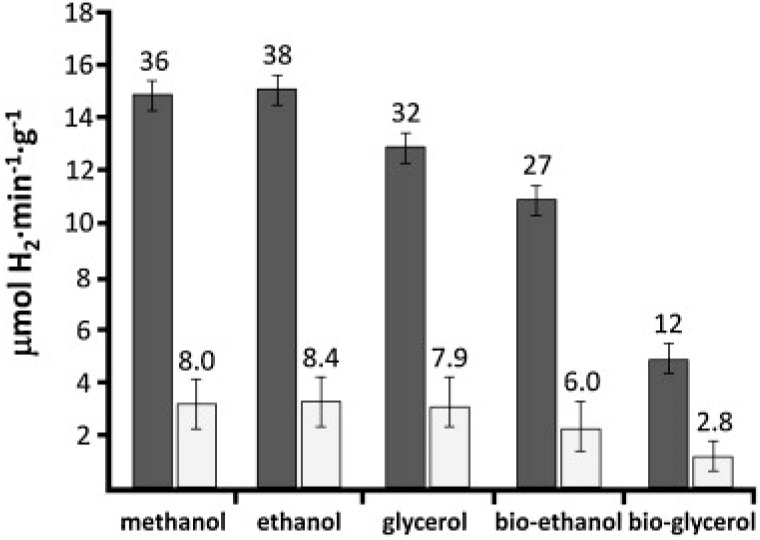
H_2_ evolution rates and quantum efficiencies of bio-alcohols (1:1 H_2_O) in a catalytic wall reactor (dark) or a slurry reactor (light) over 1 wt % Au/TiO_2_. (reproduced from [297] with permission of Elsevier).

Photoreforming *per se* has been extended to ethanol [290,291,292,293,294], various alcohol mixtures [35,294,295,296,297,298], glycerol [33,55,56,289,299], sugars [300,301], and acetic acid [302]. In the last case, no CH_4_ product was seen, an encouraging result in view of previous claims of a novel Photo-Kolbé process over a similar catalyst [303]. All these studies used TiO_2_-supported precious metals responding only to UV light at high efficiency. For 80% aqueous ethanol over a well-dispersed Pt/TiO_2_ film at low light intensities (I_uv_ ≈ 0.8 mW/cm^2^, or 0.2 suns), a remarkable quantum efficiency (Φ_uv_ ≈ 74%) was estimated [294]. Close to solar intensities, these were rather lower (Φ_uv_ = 10%–30%) for ethanol [290,295,297], but several groups have reported mass-specific H_2_ evolution rates of technical interest (>2 mmol/h/g_cat_) [33,291,293]. As shown in Figure 24 and Figure 25, bio-oxygenates are reformed at comparable rates [297,298], while quantum efficiencies can be raised significantly (to Φ ≥ 30%) due to the superior illumination geometry available in an optical fibre honeycomb reactor [297].

Early investigations of photo-reforming over visible-active catalysts include SPR metals on TiO_2_ [304], Cd_1-x_Zn_x_S/ZnO [305], CdS/TiO_2_ [306], ε-Fe_2_O_3_ [307,308], and a potentially self-degrading glucose-TiO_2_ charge transfer complex [309]. However, only the chalcogenide-based system gave a respectable quantum efficiency, Φ_>420nm_ = 9.6% [305]. Glycerol photoreforming over earth-abundant co-catalysts CuO_x_/TiO_2_ [310,311] and NiO_x_/TiO_2_ [312] has also been reported.

## 6. Photoreduction of Carbon Dioxide: Artificial Photosynthesis

As mentioned in the *Introduction*, a valuable (solar) energy-storing artificial photosynthetic process which couples two stages in the energy scheme is methanol synthesis from carbon dioxide and water:

CO_2_ + 2 H_2_O ↔ CH_3_OH + 1.5 O_2_ (23)

although ethanol synthesis:

2 CO_2_ + 3 H_2_O ↔ C_2_H_5_OH + 3 O_2_ (24)

would be even better due to its higher energy density, lower toxicity and volatility. However, Equation (24) is probably unrealistic due to its mechanistic complexity and associated selectivity issues, at least as reported in CO_2_ hydrogenation [26,27]. Methanol synthesis *per se* (Equation (23)) is already complex as it subsumes the water splitting process (see Equation (6)) but is 50% more demanding energetically (∆H = +727 kJ/mol) due to the evolution of proportionately more O_2_, as shown by the relevant half-cell reactions:

CO_2_ + 6 H^+^ + 6 e^−^ → CH_3_OH + H_2_O [E° = −0.32 V] (25)

3 H_2_O → 1.5 O_2_ + 6 H^+^ + 6 e^−^ [E° = +1.23 V] (26)

and comparison of Equations (6) and (26). While this 6 e^−^ process can be driven by near-IR photons, it is mechanistically more complex than water splitting as two elementary steps (proton reduction and H atom coupling) at the photocathode are replaced by activation of a stable gas molecule of low aqueous solubility, its multi-step reductive de-oxygenation, and progressive hydrogenation (Equation (25)). It perhaps comes as no surprise that carbon-based solar-to-fuel conversion efficiencies lag far behind those of water splitting at < 1%, with turnover rates (10–100 μmol/h/g_cat_) over pristine semiconductors still too low for technical exploitation [43,45,79]. Achieving high selectivity to methanol is also a challenge insofar as CH_4_ is preferentially obtained over hydrated anatase TiO_2_ [313,314], unless the surface Ti^4+^ (electron trap) centre is highly-dispersed or isolated [315].

**Figure 26:**
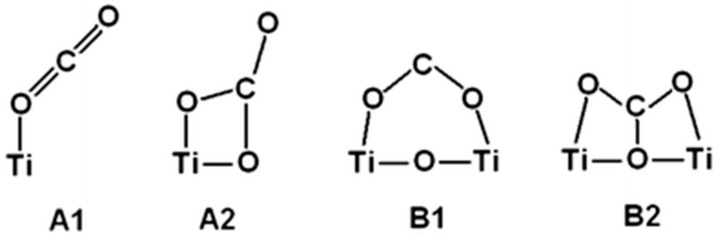
DFT-modeled states of neutral/anionic CO_2_ adsorbed on TiO_2_ anatase (101) (reproduced from [316] with permission of the Royal Society of Chemistry).

**Figure 27:**
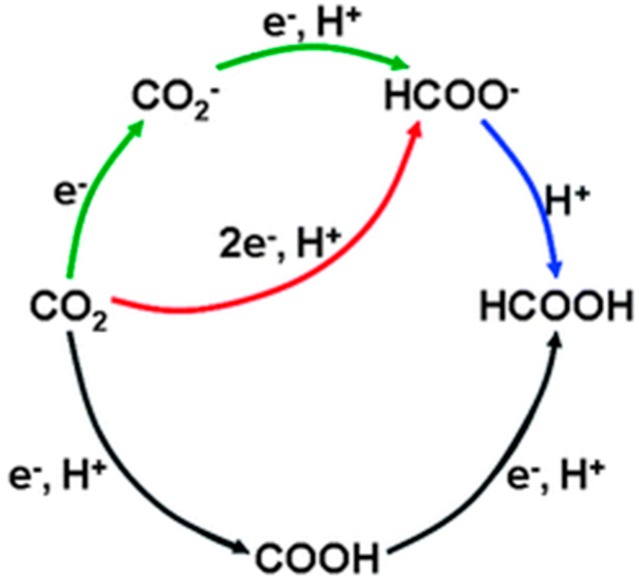
Pathways of 2e^−^/2H^+^ photoreduction of CO_2_ to formic acid. DFT energy barriers (<1 eV) favour the green route (via B1) and the red route (via A1) (reproduced from [316] with permission of the Royal Society of Chemistry).

Nonetheless, as shown in Figure 26, there is general agreement that the photocatalyst plays an indispensible role in activating CO_2_ (the probable rate-determining step) by electron transfer and stabilizing the highly energetic CO_2_**·**^−^ radical ion in coordinated form(s) [43,316,317]. Possible sequences of proton-coupled electron transfer in CO_2_ conversion to formic acid are shown in Figure 27. DFT-modeled energy barriers favor the green route via bidentate coordination mode B1 (0.87 eV) and the red route via the linear monodentate mode A1 (0.82 eV) although this is less likely as it requires a simultaneous two e^−^ transfer. Routes (black) via carbonato-type complexes A2 and B2 proceeding through carboxyl (COOH) intermediate have much higher energy barriers, 2.25 eV and 1.73 eV, respectively.

**Figure 28:**
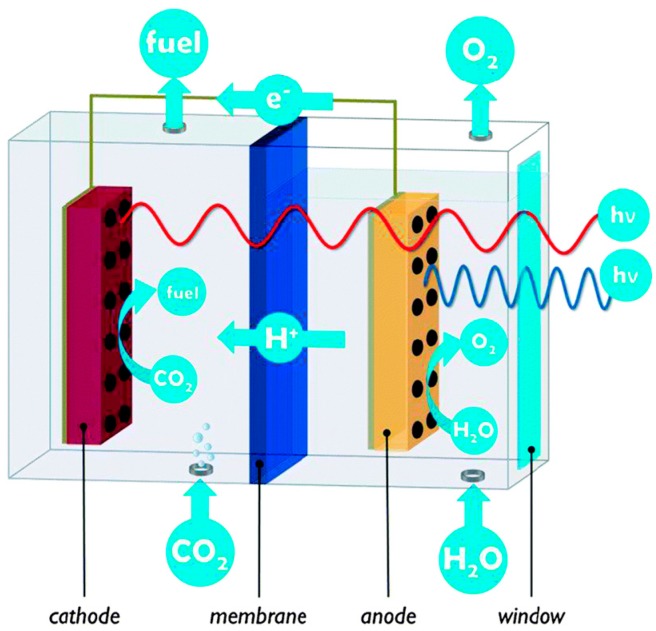
Liquid-phase 2-compartment PEC cell for CO_2_ photo-reduction. (reproduced from [79] with permission of the Royal Society of Chemistry).

It is evident from the literature [24,43,317] that earth-abundant Cu is a ubiquitous co-catalyst in CO_2_ reduction, yielding mainly methanol in gas-solid photocatalysis or methane by electrocatalysis. This selectivity effect has been rationalized [318] as due to reaction of the surface-bound methoxy (CH_3_O-Cu) intermediate either with a co-adsorbed H atom (favouring CH_3_OH by a lateral surface mechanism) as in the industrial synthesis [319], or with a proton from aqueous solution (favouring CH_4_ via attack on the protruding CH_3_ moiety). The prevalence of Cu (or CuO_x_) in composite photocatalysts [41,43,44,45,320,321,322,323] and electrodes [319,324,325,326] suggests that the dark mechanism “post-formate” is still operative but in which water photo-oxidation provides the electron/proton pairs in Equation (25), *i.e.*, the half-cell equivalents of H atoms from H_2_ dissociation. If this is the case, the thermal mechanism remains important [19] and an approach toward industrial synthesis conditions is worthy of study, e.g., mild heating/pressurization of CO_2_/H_2_O vapour under illumination. Publications have recently proliferated on the role of nanocarbons and graphenes as cocatalysts in composite photocatalysts [327,328,329], including photo- [40,330,331,332] and electro-reduction of CO_2_ [333]. This is linked mainly to their effectiveness in promoting charge separation [327,328,329,334], although there are tentative claims for visible sensitization, possibly due to adventitious C doping [40,327,328,329]. There is also growing evidence that graphene oxide can act as a photocatalyst *per se* [335] although CH_3_OH synthesis activity is improved by addition of Cu [336] and/or molecular sensitizers [337]. Otherwise, visible response has been conferred to TiO_2_ nanocomposites by incorporating CdS/Bi_2_S_3_ [338], CdSe quantum dots [339], or plasmonic metal deposits [340]. While promising advances have been made in “self-biasing” particulates, the tendency of methanol to act as an efficient hole scavenger may ultimately militate against a gas-phase photocatalytic approach in favour of a PEC (membrane-separated photo-electrode) arrangement, as shown in Figure 28. This is despite the known limitations of the latter, viz., CO_2_ solubility/mass transfer issues in the liquid phase [79], and a higher probability of obtaining CH_4_ on a photoelectrode [318,326].

**Figure 29:**
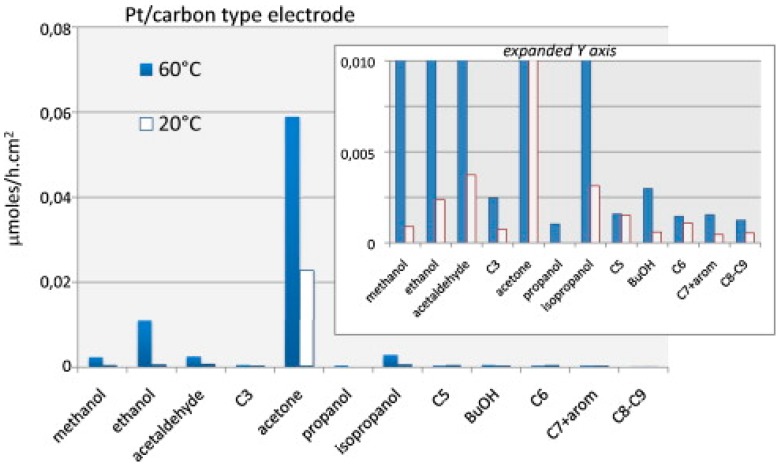
Temperature effect in gas phase CO_2_ electro-reduction on 10% Pt/Carbon/ Nafion117 GDM electrode (reproduced from [333] with permission of Elsevier).

As a potential solution, and borrowing concepts from PEM fuel cell technology, gas-phase electrocatalytic studies using a gas diffusion membrane electrode (GDM) configuration have given improved CO_2_ conversion rates (Faradaic efficiencies) and a selectivity shift towards oxygenates over carbon nanotube-supported Pt and Fe [333,341,342], as shown in Figure 29. The beneficial effect of mild heating is also evident. 

Regarding the dark mechanism in industrial methanol synthesis (from CO_2_/CO) over supported Cu, a recent microkinetic/DFT modeling paper [343] casts doubt on the formate (HCOO) → dioxomethylene (H_2_COO) route in favour of carboxyl (COOH) → formic acid [HC(OH)O] → hydroxymethoxy [H_2_C(OH)O] → formaldehyde (hydroxyl) [CH_2_O (OH)] → methoxy (CH_3_O). The hydroxymethoxy species is rightly considered the key intermediate but its identity as the conjugate base of methanediol or methylene glycol (formaldehyde hydrate) has seemingly not been recognized [343,344]. This species is implicated in the Cannizzaro disproportionation involving a hydride or proton-coupled 2 e^−^ transfer reaction [345,346,347,348]:

2 H_2_C(OH)O → CH_3_O + HCOO + H_2_O (27)

thereby providing a simpler (direct) path to methoxy.

## 7. Summary and Outlook

From this brief overview of a burgeoning field of research over the last two to three decades, it is somewhat puzzling and disappointing to admit that a “solar fuels” industry is still no more than a futuristic concept [349] with no clear indication as to when to expect its realization [58], or at least the emergence of (pre-commercial) demonstration systems. A recent review of the proliferating patent literature reveals that they are almost exclusively based on incremental advances at the fundamental level, many being filed by academic scientists [42]. However, looked at *sub specie aeternatitis,* one must recognize the enormity of the challenge facing Mankind, viz., a paradigm shift towards a sustainable global economy based on an entirely new foundation, or what could be termed “renewable petrochemistry” [350]. The expanding role of industrial catalysis in such a future will require a thorough evaluation of scalability issues, not least in global elemental resources [68] for a sector established largely on the exploitation of rare metals. Photocatalysis and the maturation of commercial photoreactor design [351] in environmental detoxification is certainly gaining an industrial foothold [13], but this reviewer has only come across one pertinent reference to H_2_ photogeneration on a pilot scale, and even this did not involve water splitting (O_2_ co-generation) but photoreforming of aqueous organic contaminants over Pt/N-TiO_2_ and Pt/CdS/ZnS [352]. Otherwise, nanoparticulate suspensions have demonstrated very low efficiencies to date (≤1%) and offer merely simplicity and convenience in operation. Disadvantages include unwieldy reactor size/catalyst charge and an associated explosion hazard in the absence of a H_2_/O_2_ separation stage. In contrast, implementation of a PEC-membrane-integrated tandem system that yields pure H_2_ at STH efficiencies already exceeding 10% in the laboratory (mandated to meet the US DOE cost target of USD 2.00–4.00 per kg H_2_ [353]), is hampered by complexities associated with device design and scale-up, especially geometric factors and their role in loss (optical and overvoltage) minimization [354,355,356]. Indeed, the question as to whether an integrated (PV-PEC) design will ultimately outperform coupled PV-electrolyzers (with independently optimized components), or if their co-development has advantages [230,233,234,357], is still open to debate [58,59,226,358,359]. Certainly the former raises more materials compatibility issues since electrolyzers work best in acid or alkaline environments. According to McKone *et al.* [58], priority needs in fundamental work include: (a) Higher efficiency electrolysis in buffered pH-neutral electrolyte (for greater durability of earth-abundant absorbers and catalysts); (b) More conductive anion (alkaline) exchange membranes (thinner separators lead to higher efficiency electrolysis and faster pH equilibration); (c) More earth-abundant OER catalysts stable in acidic media (a major expansion in PEM-based devices will otherwise be limited by their present dependence on precious metal catalysts); (d) Optical transparency in lower mass-specific activity co-catalysts (minimal parasitic light absorption); and (e) A wider range of stable (acid/alkali -resistant) visible light absorbers and transparent (ultra-thin protective) layers for Si.

Two excellent articles have appeared recently that address the technoeconomic feasibility of centralized facilities for solar hydrogen [60] and the more complex case of solar methanol, which includes the problem of CO_2_ sourcing [231]. Pinaud *et al.* [60] considered four types of reactor systems of increasing complexity designed to generate 1 ton per day of pure H_2_ at 20 bar from 0.1 M KOH solution/electrolyte. Type 1 was an array of 18 shallow plastic “baggies” (323 × 12 × 0.1 m) housing a single-bed photocatalyst particle suspension working at an assumed STH efficiency (η) of 10% (theoretical η_max_ ≈ 23%). Type 2 was similar but had two (types of photocatalyst) beds for separate generation of H_2_ and O_2_ coupled by redox mediators (Z-scheme)—η = 5% (η_max_ ≈ 15%). Type 3 was a fixed panel array of ~27,000 monolithic tandem absorber PEC cells—η = 10% (η_max_ ≈ 30%), while Type 4 was similar but with a drastically reduced number of cells (~2,000, generating equivalent power) due to coupling with a tracking concentrator (×10) assembly—η = 15%. The estimated cost of H_2_ (per kg) for the four cases was $1.60, $3.20, $10.40, and $4.00, respectively. These are encouraging figures but it should be recognized that the efficiencies assumed for the particulate systems were “target” values several times higher than the current state-of-the-art. Clearly, the most promising and realistic system for early demonstration would be Type 4. However, the inclusion of low-power concentrators adds further complexity [360] and raises the question as to how PEC-based systems actually respond to light intensification and to what extent it is influenced by device configuration. While it is well-known that efficiencies in PV arrays are maintained (or even improved) by concentration of sunlight [361,362], the progressive drop in photochemical quantum yields with increasing incident power in suspended particulate systems is notorious [363], explaining the virtual absence of concentrating optics in advanced oxidation photoreactors, excepting perhaps the compound parabolic reflector for improved collection of diffuse sunlight [351]. The problem is generally attributed to kinetic limitations in surface redox processes and/or O_2_ supply due to low aqueous solubility. Some of these “chemical” constraints are shared by PEC cells, such that a strong case can be made for a systematic evaluation of the sensitivity of water splitting efficiency to solar light concentration. The only positive evidence in the open literature is the NREL claim of a record-breaking 12% efficiency (in 1998) using a prototype hybrid PV/PEC system comprising a GaInP_2_ photocathode voltage-biased by an underlying GaAs (PV) absorber under 11 Suns illumination [364].

**Figure 30:**
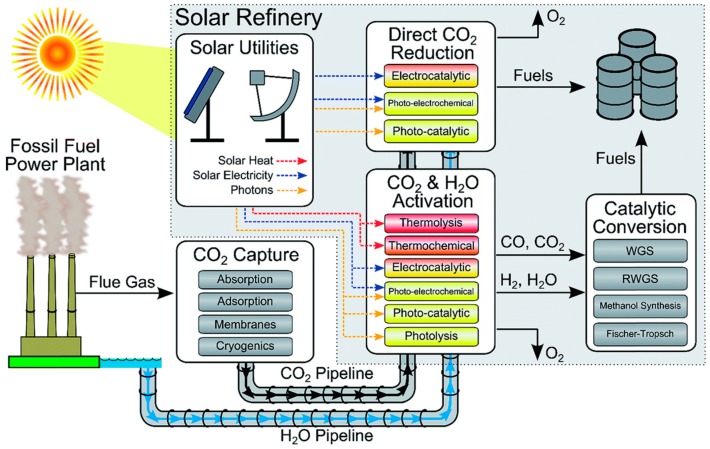
Scheme of a solar refinery based on CO_2_ reduction to methanol *indirectly* by renewable H_2_ (ex solar photon- or heat-driven electrolysis) or *directly* (“one pot”) with H_2_O using solar-electricity (PEC) or solar photons (photocatalysis) (reproduced from [231] with permission of the Royal Society of Chemistry).

Herron *et al.* [231] have taken a broader approach in designing a “transitional” solar refinery that produces H_2_ as an intermediate in the generation of liquid fuels but with sub-systems still dependent to some degree on fossil fuel energy input, as represented schematically in Figure 30. First, a feasibility study was made by assessing the energy balance for the *indirect* route based on existing (sub-system) efficiencies. The CAMERE process [365] was selected and modeled, viz., methanol synthesis (at 1 kg/s ≡ 22.7 MW_HHV_) from CO_2_ (captured from a fossil fuel power station) with H_2_ (produced increasingly from solar energy), and defining the solar or primary “energy incorporation efficiency” (EIE) as being positive (viable) only when the methanol energy content exceeded the sum of all fossil energy inputs. This rather severe criterion was not satisfied even when all the H_2_ was derived from solar energy. Thus, there is a need for diversification and greater implementation of solar technologies in early demonstration systems. A good candidate (energy-intensive) process for fossil fuel substitution is solar heat-driven CO_2_ recovery in amine scrubbers and, ultimately, atmospheric trapping devices [20,21].

**Figure 31:**
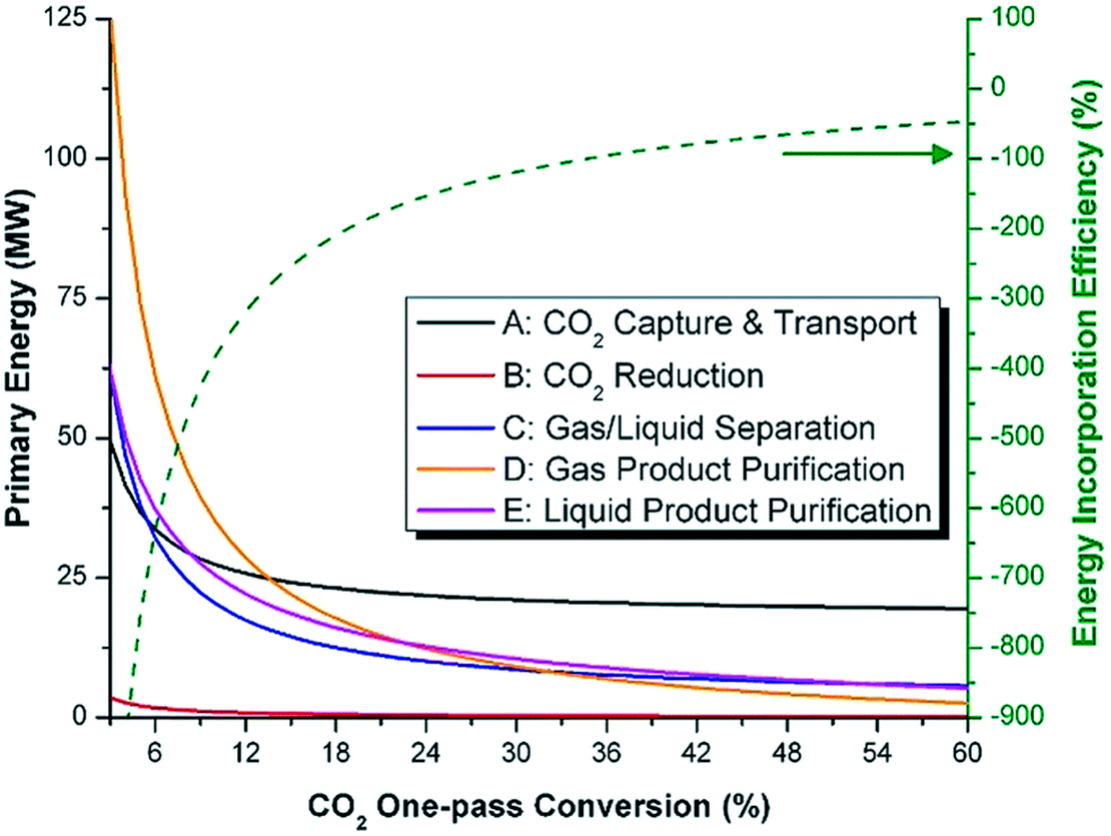
Dominance of CO_2_ capture stage on primary energy costs in photocatalytic CH_3_OH synthesis from CO_2_/H_2_O (reproduced from [232] with permission of the Royal Society of Chemistry).

**Figure 32:**
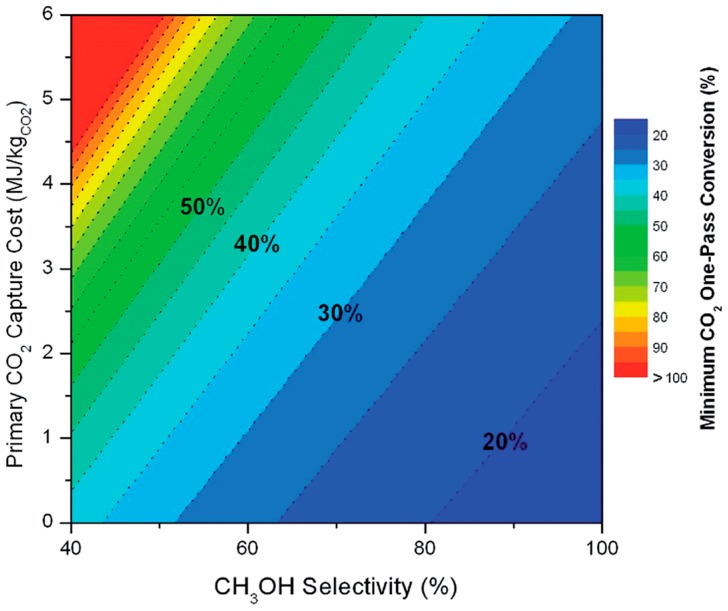
Effect of photocatalyst selectivity and CO_2_ capture cost on minimum one-pass conversion for positive energy incorporation efficiency (EIE > 0) (reproduced from [231] with permission of the Royal Society of Chemistry).

A case study estimating the threshold CO_2_ single-pass conversion for positive EIE based on a *direct* (photocatalytic) route was also made, assuming a reaction selectivity of 40% as below:

CO_2_ + 2 H_2_O → 0.6 CH_4_ + 0.4 CH_3_OH + 1.8 O_2_ (28)

and a catalyst mass-specific rate of ~1 μmol/g_cat_/h (Φ_uv_ = 0.28% over Ti-containing porous SiO_2_ [366]). As shown in Figure 31, it is primarily the CO_2_ capture stage (from dilute flue gas) that impedes the energy efficiency break-even point (EIE = 0), such that present costs of around 5.5 MJ/kg_CO2_ must be further reduced. If the enthalpy from burning the renewable CH_4_ co-product is valorized, positive energy incorporation is feasible below 50% conversion. Better still, if a more selective catalyst can be developed, it would simplify the process and have a dramatic effect on minimum one-pass conversion (EIE > 0), as shown in Figure 32. However, by the authors’ own admission, this case is quite impractical due to the low activity of the photocatalyst, roughly 3 orders of magnitude below that required as discussed in Section 4 and Section 5. Even at 1 mmol/g_cat_/h, for an overall production rate of 1 kg/s, it would need 100 metric tons of catalyst and a reactor volume of 100 m^3^ severely constrained in one dimension by optical factors.

In closing, the author feels obliged to point out that the earlier review [19] was written on the premise that future market penetration by the renewable energy sector would be substantial and already impacting (slowing the rise of, if not stabilizing) global CO_2_ levels in the atmosphere. Unfortunately, governments have not provided sufficient incentives or regulatory measures to curb our dependence on fossil fuels and the oil industry has conducted very much a “business-as-usual” policy. In the last 20 years, the rate of emissions has increased by 50% and a cumulative level of 400 ppm CO_2_ has been reached, *i.e.*, 50 ppm in excess of the threshold considered necessary to avoid a mean global temperature rise of more than 2 °C with probable catastrophic effects. While the penetration of CO_2_-neutral energy systems will help to ease the burden on natural sinks for CO_2_, it now appears essential to augment these with artificial (man-made) disposal methods. The author’s directions in research have broadened accordingly in recent years, favouring mineralization as the only technology that will guarantee CO_2_ sequestration on the requisite (geological) time-scale [367,368,369,370,371].
